# Selective cultural adoption: The roles of warmth, competence, morality and perceived indispensability in majority‐group acculturation

**DOI:** 10.1111/bjso.12801

**Published:** 2024-09-16

**Authors:** Jonas R. Kunst, Kinga Bierwiaczonek, Milan Obaidi, Sam Fluit, Tilmann von Soest, David Sam, John F. Dovidio

**Affiliations:** ^1^ Department of Psychology University of Oslo Oslo Norway; ^2^ Department of Psychology University of Copenhagen Copenhagen Denmark; ^3^ Department of Psychosocial Science University of Bergen Bergen Norway; ^4^ Department of Psychology Yale University New Haven United States

**Keywords:** acculturation, competence, immigrants, intergroup relations, majority acculturation, morality, perceived indispensability, warmth

## Abstract

Psychological research has begun considering the dynamics involved in majority‐group acculturation, which is the extent to which cultural majority groups adopt the culture of immigrants and minority groups. However, previous research has predominantly concentrated on reactions to ‘immigrants’ or ‘minority groups’ as a homogenous entity, overlooking the nuanced perceptions and varied valuations attributed to different groups. Recognizing the heterogeneity among immigrant and minority groups, the present work investigated the influence of several perceived characteristics of immigrant and minority groups on majority‐group members' adoption of their cultures. Specifically, in three pre‐registered studies—one correlational (*N*_participants_ = 201, *N*_trials_ = 2814) and two within‐subjects experimental (*N*_participants_ = 144 and 146, *N*_trials_ = 720 and 730) designs with close to politically representative samples from the U.K. and U.S.  —majority‐group members were more willing to adopt immigrant and minority‐group cultures that they perceived as warm, competent and moral because these perceptions made immigrants and minority groups seem indispensable to the identity and economy of the mainstream society. Our studies highlight the importance of considering the differentiated acculturation that majority‐group members have to various groups within the same national context. We discuss the societal and cultural repercussions of this selective uptake of other cultures.

## BACKGROUND

While research on social influence traditionally focused on the influence of the majority on the minority, minority‐group members can exert influence on the thoughts and actions of majority‐group members as classic (Moscovici & Nemeth, 1974) and more recent research has revealed (see Butera et al., 2017, for a review). This social influence holds particular significance in the field of acculturation, which is dedicated to exploring the dynamics of cultural transmission and change. Although acculturation is commonly defined as a two‐way process, research has only recently focused on the cultural and psychological changes experienced by ethnic majority‐group members living in increasingly diverse societies (Kunst, Lefringhausen, Sam, et al., 2021). Previous work has frequently studied the preferences of majority‐group members concerning how immigrants and other minority‐group members should acculturate (i.e., ‘acculturation expectations’; see Montreuil & Bourhis, 2004) or how diversity should be managed at the societal level (e.g., ideologies such as ‘multiculturalism’ or ‘interculturalism’; Verkuyten & Thijs, 2002; Yogeeswaran et al., 2021). By contrast, majority‐group acculturation refers to the extent to which members of a socially dominant group adopt aspects of the culture of immigrant and other minority groups and/or maintain their dominant culture (Kunst, Lefringhausen, Sam, et al., 2021).

In the current research, we adopt the definition of an immigrant as ‘a person who moves into a country other than that of [their] nationality or usual residence, so that the country of destination effectively becomes [their] new country of usual residence’ (International Organization for Migration, 2019, p. 103). A minority group is defined as ‘a group numerically inferior to the rest of the population of a State and/or in a non‐dominant position, whose members possess ethnic, religious or linguistic characteristics differing from those of the rest of the population’ (International Organization for Migration, 2019, p. 144). As these definitions show, both groups can overlap. Ethnic minority‐group members can consist of current immigrants or their descendants but also of traditionally marginalized indigenous people.

At first glance, the idea that majority‐group members acculturate towards the culture of immigrants and minority groups may seem counterintuitive. After all, they belong to the dominant culture in society and experience less pressure and practical incentives to change their culture. However, each encounter with newcomers provides the opportunity to learn from and improve one's cultural repertoire (see Molleman et al., 2013). History is full of examples where various types of newcomers transformed the majority societies they joined. The Vikings and Huguenots influenced the English language, art, architecture and values. Chinese immigrants contributed to Canada by introducing new cuisine, music, dances and martial arts. Jewish immigrants transformed U.S. American literature, music and film. Such changes to the fabric of societies' mainstream culture are only possible if many majority‐group members are receptive to and adopt new cultural content (also see Erten et al., 2018).

Indeed, previous studies show that across a range of immigrant‐receiving societies, a sizable group (about 30%) of majority‐group members report that they generally adopt the culture of immigrants (Kunst, Lefringhausen, Sam, et al., 2021). At the same time, a group of similar size tends to reject immigrant cultures, whereas the remaining cluster is undecided. Some studies have examined the underlying personality dimensions (e.g., openness to experience) or social‐psychological factors (e.g., ethnocentrism, national or global identity) among majority‐group members that shape their acculturation orientations towards ‘immigrants’ or ‘minority‐group members’ generally (Haugen & Kunst, 2017; Komisarof, 2009; Kunst, Lefringhausen, Skaar, & Obaidi, 2021; Lefringhausen et al., 2021, 2022; Lefringhausen & Marshall, 2016; Ozer et al., 2021). However, while offering some general insights into the majority populations' overall acculturation orientations and the influence of individual characteristics, a significant limitation of this existing research is that it mostly has assessed these orientations generically without specifying the cultural group in question or considering its characteristics. Because societies usually consist of many immigrant and minority groups that are assigned different statuses and prestige by the majority society, majority‐group members can be expected to adopt the culture of some groups more than others.

Addressing this gap, we propose and aim to empirically demonstrate in three pre‐registered studies that the distinct ways groups are perceived on key dimensions of intergroup evaluations (e.g., warmth, competence, morality; Leach et al., 2007) explain majority‐group members' selective cultural uptake of these groups' cultures. Furthermore, we investigate whether a fundamental rationale for the observed effect of these intergroup perceptions on heightened cultural adoption is their role in enhancing the perceived functional‐ and identity‐related indispensability of immigrants within society (Ng Tseung‐Wong & Verkuyten, 2010). For example, the perception of warmth may result in a greater perceived indispensability related to societies' social identity, or perceptions of competence may lead to an increased sense of economic (or functional) indispensability. These indispensability perceptions, in turn, could elevate the value attributed to adopting the respective group's culture. To demonstrate this, we experimentally manipulate the entire mediation pathway (intergroup perceptions → perceived indispensability → culture adoption). In the following sections, we outline our conceptual model's theoretical and empirical basis.

### Key dimensions of intergroup perceptions

Seminal research (Fiske, 2018; Fiske et al., 2002) demonstrated the robust impact of two fundamental dimensions, warmth and competence, in shaping people's perceptions of social groups and their members. Warmth refers to how friendly, trustworthy, and likable a group is perceived to be, whereas competence refers to how capable, skilled and successful it is believed to be. Both dimensions consistently predict attitudes towards various groups, including immigrants (Fiske, 2018). Moreover, the extent to which people view groups as warm and/or competent has distinct consequences also for intercultural relations. For instance, higher perceptions of competence and warmth of an immigrant group predict generally more positive attitudes towards the group and more welcoming acculturation ideologies (i.e., less assimilationism, more multiculturalism; Kil et al., 2019).

In a different set of seminal studies, Leach et al. (2007) showed that the perception of social groups as moral (e.g., honest, sincere) explained positive attitudes towards them over and above the effects of competence and warmth. More recent studies suggest that the three dimensions of intergroup perceptions are complementary, each explaining a share of variance in out‐group evaluations (Constantin & Cuadrado, 2021; López‐Rodríguez, Zagefka, et al., 2014).

We hypothesized that these three fundamental dimensions of intergroup perceptions–warmth, competence and morality–would also play critical, independent roles in majority‐group members' acculturation orientations. First, warmth is related to less perceived competition and more positive emotions (Bye et al., 2014; Constantin & Cuadrado, 2021; Froehlich & Schulte, 2019; López‐Rodríguez, Navas, et al., 2014). Thus, if immigrants are seen as warm and approachable, this may create a more welcoming environment for cultural exchange. Moreover, a perceived lack of warmth of immigrants is related to an assimilation‐like expectation towards them (Kil et al., 2019; Urbiola et al., 2021), which is logically opposite to viewing acculturation as a two‐way process, wherein all groups in contact undergo change.

We further predicted that when immigrants are perceived as competent, majority‐group members may be more motivated to adopt their culture. Adopting the culture of groups that are perceived as competent may be an opportunity for growth and increased success of the in‐group and its members (Kunst & Mesoudi, 2024; Mesoudi, 2011). Indeed, majority‐group members are less likely to exclude and more willing to cooperate with immigrants perceived as competent (Froehlich & Schulte, 2019).

Lastly, we expected that the perceived morality of immigrants would predict majority‐group members' cultural adoption. If immigrants are viewed as morally upstanding, majority‐group members might be more inclined to trust them and consider their cultural practices inherently valuable and worth adopting. Consistent with this perspective, perceiving immigrants as moral has been linked to less negative and more positive intergroup evaluations and reactions (Constantin & Cuadrado, 2021; Cuadrado et al., 2021). Majority‐group members also are more supportive of immigrants maintaining their culture and more willing to behaviorally oppose the discrimination they may experience if immigrants are perceived as moral (Brambilla et al., 2013; Urbiola et al., 2021).

We also tested for interactive effects between warmth and competence, such that culture adoption is highest when immigrant groups are simultaneously perceived as competent and warm. This prediction is consistent with previous findings and theoretical notions suggesting that out‐groups are most positively received when they are seen as both warm and competent (Fiske et al., 2002; Lee & Fiske, 2006). We further examined interactions of morality with warmth and with competence and also tested for the three‐way interaction involving the three dimensions. These analyses were exploratory: We had no theoretical or empirical basis for testing these interactions but wanted to maximize insights into the data.

### The mediating role of perceived indispensability

Beyond increasing the understanding of majority‐group acculturation by studying the differentiated influence of perceived immigrant group variation on fundamental dimensions, the current research extends previous work by examining a key mediator, perceived indispensability (Guerra et al., 2015; Verkuyten et al., 2014). Whereas much research on host‐immigrant relations has identified intergroup threat of various forms (e.g., realistic/economic and symbolic/identity) as key factors (Stephan et al., 2016), orientations towards immigrants and minority groups may also be driven by positive elements of the relationship as are other forms of intergroup relations. For instance, in Sherif et al.'s (1961) classic Robber's Cave studies, two groups of boys in competition and conflict developed more reciprocally positive and productive relations when they had superordinate goals – aspirations that could only be achieved by the joint contributions of both groups and the reciprocal appreciation of each group's indispensability. Applied to a migration context, perceived indispensability refers to the extent to which immigrants are viewed as integral to the economic fabric and identity of the host country by majority‐group members (Guerra et al., 2015; Mepham & Verkuyten, 2017; also see Walsh & Tartakovsky, 2020). If placed on a spectrum, indispensability perceptions could be conceptualized as the opposite endpoint of threat perceptions, with neutral perceptions positioned at the midpoint. However, it is important to note that it is possible for groups and individuals to be perceived high in indispensability in one domain (e.g., technology, STEM) but as a threat in another (e.g., espionage).

These indispensability perceptions typically fall within two distinct, yet often strongly and positively correlated dimensions (Guerra et al., 2015, 2016): *Identity indispensability* pertains to the perception that immigrants constitute a vital component of a country's cultural identity (Fluit et al., 2023; Verkuyten & Khan, 2012), whereas *economic indispensability* – which is a form of functional indispensability – relates to the belief that the absence of immigrants would be detrimental to the economy (Mepham & Verkuyten, 2017). In terms of the latter economic indispensability and in addition to identity indispensability, our research narrows its focus on the perceived economic indispensability of immigrants rather than their other comprehensive functional roles. We therefore opt for the term perceived ‘economic indispensability’ throughout, while recognizing that this concept is a particular variant of the wider concept of 'functional indispensability.'

#### The potential effects of warmth, competence and morality on indispensability

In classical research on the stereotype content model, perceptions of status and competition were considered to shape stereotypes (Fiske et al., 2002). For example, groups viewed as higher in status were stereotyped as more competent, and those seen more as competitive were perceived to be less warm (see Cuddy et al., 2009). However, as further proposed in the stereotype content model, stereotypes are highly consequential, shaping perceptions, attributions and emotional responses and actions towards a group and its members. Stereotypes thus exert significant downstream effects in social evaluative processes because they convey socially relevant content about groups and individuals (Yzerbyt & Demoulin, 2010).

Indeed, experimental research supports the bi‐directional causality of the process described above, where groups described as more competent are perceived as having higher status (Durante et al., 2010), a causal direction acknowledged by the founders of the stereotype content model early on (Fiske et al., 2002). Of particular relevance to the goals of our research, perceptions of workers on dimensions such as competence influenced how their contributions to organizations were perceived (Cuddy et al., 2011). This leads us to the possibility that perceptions of high levels of warmth, competence and morality could potentially inform perceived indispensability. For instance, from a social identity perspective (Tajfel, 1978), including a group recognized as warm, competent and/or moral can aid majority‐group members in fortifying a favourable in‐group identity, thus contributing to the perceived identity indispensability of immigrants. Moreover, it could enhance the perceived significance that immigrants have to the economy (i.e., their economic indispensability), in line with research showing the impact of intergroup perceptions on evaluations in work‐related settings (Kunst, Kirkøen, & Mohamdain, 2023).

##### Warmth

Whereas each of the three dimensions of intergroup evaluation may impact the two forms of perceived indispensability of immigrants, their influence might be particularly pronounced in specific areas. Warmth primarily reflects the degree to which individuals are expected to be empathetic and cooperative and prioritize the group's interests over individual interests (Cislak & Wojciszke, 2008). It thus constitutes an essential aspect for the formation of functional social groups (Brewer, 1999; Van Vugt & Hart, 2004). Previous research has linked warmth with the process of integrating oneself into a group of strangers, thereby forming a social identity, while the link with competence was found to be non‐significant (Kong, 2018). Hence, it might be posited that warmth predominantly influences perceptions of the identity indispensability of immigrants.

##### Competence

Competence pertains to attributes such as the perceived skillfulness and intelligence of a group or individual, and is particularly salient in social evaluations in organizational and economic contexts (Cuddy et al., 2011). Therefore, perceived competence may exert a particular impact on economic indispensability, signifying the inherent value that newcomers bring to an economy, for instance because highly competent immigrants can fulfil critical roles with high recruitment demand, such as specialists.

##### Morality

Morality is posited to play a pivotal role in shaping people's social identities and in the delineation of individuals as either insiders or outsiders (Ellemers et al., 2017). Moral character is significant in impression formation because it reflects the nature of a person's intentions and whether those intentions are geared towards being beneficial or detrimental, virtuous or malicious (Goodwin, 2015). Indeed, although all three dimensions have been demonstrated to predict trust – a factor integral to both types of perceived indispensability – morality tends to have the most substantial impact (Weiss et al., 2021). Thus, perceived morality can be expected to play a similar role in both perceived economic and identity indispensability. It signals the trustworthiness of prospective group members, which indicates whether they will be reliable group members and contribute to social identity cohesion. Morality is also of great significance for perceived economic indispensability, as it may indicate a sincere intention to contribute economically and the absence of tendencies to exploit collective resources without contributing.

#### Perceived indispensability and majority‐group acculturation

Importantly, these perceptions of indispensability could subsequently influence the acculturation orientations of majority‐group members in systematic ways. The perception of indispensability diminishes social distancing – an obstacle to intercultural interaction and, by extension, acculturation (Guerra et al., 2015). Concurrently, it triggers the activation of shared superordinate group categorization, which improves attitudes, increases valuing and promotes acceptance (Gaertner et al., 2016), thereby likely fostering acculturation (Guerra et al., 2015; Verkuyten et al., 2014). If majority‐group members perceive immigrants as belonging to the same overarching group, they are more likely to see both groups as sharing similarities (Gaertner et al., 1993; Gaertner & Dovidio, 2000; Kunst et al., 2015). This perception of similarity is a well‐established factor that modulates the influence of intercultural contact on acculturation (Schwartz et al., 2010). Especially if this common group identity accommodates additional identities, such as dual identities, and thus encourages identity complexity, it may enable immigrant groups to assume specialized roles and functions, while also being part of a shared group, thereby increasing their indispensability to the nation. Moreover, the positive appraisal of an immigrant group's contributions is correlated with increased interactions with the group (Tartakovsky & Walsh, 2022). This interaction is fundamentally vital for acculturation, offering further theoretical support for the predicted role of indispensability perceptions in the acculturation of the majority group.

### The present research

The aims and potential contributions of this research are multifaceted. The influence of minorities has been explored for decades within social groups defined by characteristics such as political affiliations or gender (Butera et al., 2017). However, to our knowledge, it has not been examined systematically within the context of ethnicity, aside from the nascent research area concerning the acculturation of majority groups, to which our work directly contributes. Given the early state of this research area, the impact of attributes perceived to differ among minority groups on the acculturation processes of majority‐group members remains largely unexplored.

Consequently, via three pre‐registered studies, we aimed to make an important theoretical contribution to this developing area by melding it with foundational frameworks related to intergroup perception and evaluation. The first study investigates the main associations within our conceptual model through a correlational approach, specifically examining the link between intergroup perceptions of warmth, competence and morality with the cultural adoption of majority groups. While this study emphasizes ecological validity by focusing on perceptions of real groups, it does not establish causality in a controlled manner. Therefore, the second study experimentally manipulated intergroup perceptions to test their influence on the proposed mediator (i.e., perceived indispensability) and the outcome (i.e., cultural adoption). As this second study is limited by testing for mediation with partly correlational data, the third and final study experimentally manipulated the mediator, perceived indispensability, to test its causal effect on cultural adoption.

Exploring the determinants that influence the selective adoption of elements of minority, immigrant groups' culture by members of the majority group is crucial for understanding cultural transformation. Beyond the field of acculturation, this topic holds significance for disciplines such as cultural evolution. On a practical level, our research has the potential to guide interventions aimed at preventing the formation of inequitable cultural hierarchies due to biased cultural transmission, thus fostering equality and the success of diverse societies.

## STUDY 1

The U.K. has a rich and complex migration history, shaped by its colonial past, economic opportunities and geopolitical events. This past has resulted in a diverse population composed of various immigrant and minority groups, each with unique backgrounds, migration histories and experiences. The status of these groups within the social hierarchy can vary significantly depending on factors such as voluntary or involuntary migration, legal status, racialization and the context of reception (Ford, 2011). For instance, long‐established groups in the U.K., like those with Irish and Indian heritage – who often but not always migrated voluntarily and legally – generally occupy a higher social status due to their longer integration period and economic contributions. By contrast, groups such as the Roma and Gypsy (or Irish Travellers) frequently face systemic discrimination and experience lower social status, partly due to their historical marginalization. Similarly, recent migrants from Eastern Europe, like the Polish and Romanians, navigate a complex reception context where legal status and economic roles as well as discrimination significantly influence their social standing (cf. Robertson, 2019). Thus, to provide a nuanced understanding of how different immigrant and minority groups are perceived within the U.K., in this first study, White British participants indicated warmth, competence and morality ratings for a diverse set of 14 immigrant groups in the U.K. and their own group. After that, participants indicated how much they adopted the culture of each group. Finally, they reported to what extent they maintained their British mainstream culture. With these data, we tested a series of pre‐registered predictions.

Based on the theoretical rationales outlined in the introduction, we investigated whether perceived warmth (H1), competence (H2), their interaction (H3) and morality (H4) would positively predict culture adoption.^1^ Next, as pre‐registered, we examined whether participants' ratings of their own culture moderated the effects of warmth, competence and morality. Here, we tested two competing predictions: (a) culture adoption is highest when immigrants and minority groups are seen as warmer, more competent, or more moral than the in‐group (H5a), or (b) culture adoption is highest when immigrants and minority groups are seen as similar to the in‐group on the three dimensions, suggesting curvilinear relationships (H5b). The former hypothesis suggests that majority‐group members are inclined to adopt the culture of immigrant and minority groups they perceive as superior, because incorporating them into the self may bolster self‐esteem and a positive group identity (Alicke & Sedikides, 2009; Sapienza et al., 2010; Sedikides & Gregg, 2008; Tajfel, 1982; Tajfel & Turner, 1979). The latter hypothesis suggests that such cultural adoption may occur if immigrant and minority groups are perceived as similar to the in‐group, a notion backed by research highlighting the appeal of similarity (Byrne, 1997; Montoya et al., 2008).

Finally, we tested whether the association between own culture maintenance and immigrant culture adoption would become more positive the more favourably the immigrant and minority groups are evaluated on the three dimensions (H6). Whether individuals perceive both acculturation dimensions (own culture maintenance and other culture adoption) as reconcilable has been tested among minority‐group members, finding an average weak and negative relationship (*r* = −.18) between them (see Yoon et al., 2020 for a meta‐analysis). However, we are only aware of one study investigating the factors moderating this relationship among majority‐group members (Kunst, Coenen, et al., 2023). Whereas this existing study focused on the role of individual differences (e.g., participants' global identity, political orientation) and the adoption of the culture of immigrants generally, we aimed to test whether positive intergroup perceptions of a range of different immigrant and minority groups would moderate the association between participants' own culture maintenance and other culture adoption. Based on the notion that people strive for a positive and integrated self‐concept (Benet‐Martinez & Haritatos, 2005; Sedikides & Gregg, 2008; Tajfel, 1982), we predicted that the more positively the respective out‐groups are evaluated, the more positively both acculturation orientations (own culture maintenance and other culture adoption) would become correlated.

### Method

#### Participants

A pre‐registered power simulation using the SIMR package (Green & MacLeod, 2016) suggested that 200 participants with each 14 responses (i.e., 2800 trials in total) would provide 90% power to detect a small to medium‐sized cross‐level interaction^2^ (*b* = 0.25) at a .05 significance level. Thus, we recruited a politically representative (as of January 26, 2023) and gender‐balanced sample of 201 participants (*M*_age_ = 43.30, *SD*_age_ = 14.63; women: 48.8%, men: 50.7%, other: 0.5%) from the U.K. via Prolific. Participants in this and the remaining studies were paid the equivalent of £8.5/hour. Detailed demographic information is presented in the Supporting Information.

#### Procedure

The present study was pre‐registered at https://osf.io/qvjky/?view_only=ac984a48a3134688a2131c41c8194e61. All materials, code and data for this and the remaining studies are available at https://osf.io/gav7r/?view_only=cd41212909444228b5e345426df61ead. Participants first completed two measures assessing their political orientation. They then indicated their intergroup perceptions and culture adoption in terms of 14 immigrant or minority groups in the U.K. and their own group: African, Arab, Bangladeshi, Caribbean, Chinese, German, Gypsy (or Irish Traveller), Indian, Irish, Italian, Pakistani, Polish, Roma, Romanian and British people. These groups were selected based on U.K. census data of the official ethnic categories (Office for National Statistics, 2022) and the most frequent immigrant groups (Office for National Statistics, 2021). Participants first rated each group's competence, warmth and morality (in random order) and then their acculturation orientations towards it. The order in which the groups were rated was randomized within the warmth, competence, morality and acculturation measures to prevent order effects. In this and the remaining studies, participants had to complete the survey using a personal computer.

The present research was approved by the ethical review board of the institution of the first author (Nr. 24672827). All participants provided informed consent.

#### Measures

##### Warmth, competence and morality

We presented participants with instructions adopted from Cuddy et al. (2009). To reduce social desirability, these asked participants to indicate how people in the U.K. (rather than they themselves) perceived the different groups. Next, participants rated in random order the different groups on three warmth items (likable, friendly, warm), three competence items (intelligent, competent, skilled), and three morality items (honest, sincere, trustworthy) adopted from Leach et al. (2007). Each item was rated on a 7‐point scale ranging from 1 (*not at all*) to 7 (*extremely*). By averaging these items, we obtained reliable warmth (*α*s = .92–.97), competence (*α*s = .90–.96) and morality scales (*α*s = .93–.97) for each group. Thus, higher scores indicate more perceived warmth, competence and morality, respectively.

##### Culture adoption

For each group, participants indicated the extent to which they found it important to adopt its culture in six domains using a 6‐item scale from Kunst, Ozer, et al. (2023); e.g., “How important is it to you to adopt the values of [group name] people in the UK?” (*α*s = .97–.98). The domains included the way of living, traditions, values, culture centrality, belonging and contact. The items were averaged so that higher scores represented more cultural adoption.

It is crucial to note that individuals less familiar with literature on acculturation might contend that the dimensions of cultural adoption and perceptions of groups are conceptually overlapping. Therefore, it was important to establish that they were factorially different. A confirmatory factor analysis with four correlated distinct factors supported this, showing a close fit to the data, *χ* ^2^(84) = 1475.91, *p* < .001, CFI = 0.956, RMSEA = 0.077, SRMR = 0.025. Additionally supporting the distinctiveness between the constructs, the stereotype factors were only weakly related to the cultural adoption factor, *r*s < .37, *p*s < .001.

##### Culture maintenance

On a 6‐item scale by Kunst, Ozer, et al. (2023) matching the domains of the culture adoption measure, participants indicated how important they saw it to maintain the majority‐group culture (e.g., ‘How important is it to you to live in accordance with British values?’; *α* = .97). All items were rated from 1 (*not important at all*) to 7 (*very important*) and averaged so that higher scores indicated more culture maintenance.

##### Attention checks

A seventh item in the culture maintenance instrument asked participants to select the fourth response option to show that they paid attention. In addition, they were asked to select ‘blue’ out of a list of colours in the demographics section. None of the participants failed both attention checks (our pre‐registered exclusion criterion). Thus, all participants were included in the analyses.

#### Analyses

The analyses in this and the remaining studies were conducted using multi‐level modelling where target groups were nested within participants in R 4.2.2 (R Core Team, 2022) using the lme4 (Bates, 2010), lmerTest (Kuznetsova et al., 2016) and jtools (Long, 2019b) packages. Effects were visualized using ggplot2 (Wickham et al., 2016), ggeffects (Lüdecke, 2018), interplot (Solt et al., 2021) and the interactions package (Long, 2019a).

### Results

#### Correlations

We first present and visualize correlations to maximize insights into the data and to provide face validity for the association between intergroup perception and culture adoption. Across the target groups, warmth was positively correlated with competence, *r*(2797_trials_) = .63, *p* < .001, *r*(201_subjects_) = .53, *p* < .001 and morality, *r*(2794_trials_) = .78, *p* < .001, *r*(201_subjects_) = .69, *p* < .001. Competence and morality were also positively correlated, *r*(2787_trials_) = .79, *p* < .001, *r*(201_subjects_) = .67, *p* < .001 (see Supporting Information for all correlations for all studies). Please note that multicollinearity cannot be assessed in the typical ways in multi‐level models as for single‐level regressions. However, the presence of both fixed and random effects can absorb some of the variance, potentially mitigating the impact of multicollinearity. Nevertheless, we address this issue empirically by experimentally manipulating the dimensions in Study 2.

**FIGURE 1:**
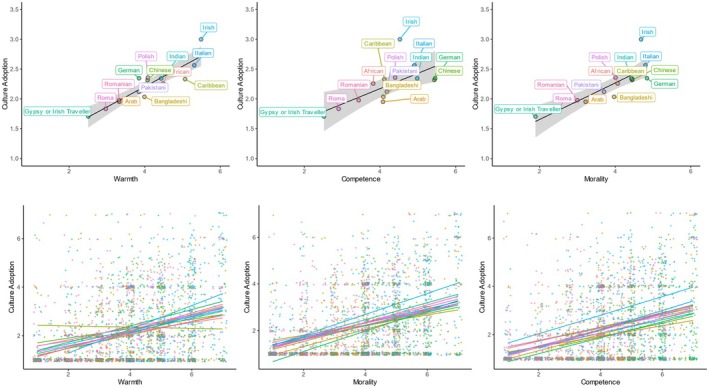
Association of intergroup perceptions and culture adoption between (top panel) and within immigrant groups (bottom panel) in Study 1. On the top panel, ribbons represent 95% confidence intervals.

Next, we visualized the correlations between each rating dimension and participants' adoption of the groups' culture at the aggregate target group and individual response level (see Figure 1). Although culture adoption was generally low, the warmer, more competent and moral a group was perceived to be, the more participants reported adopting its culture.

#### Modelling the unique and interactive associations of warmth, competence and morality with cultural adoption

We set out to test our hypotheses. As predicted in H1, H2 and H4, warmth, competence and morality were each uniquely associated with more culture adoption in our first model, *Akaike information criterion* (AIC) = 6951.39, *Bayesian Information Criterion* (BIC) = 7052.24, *R* ^2^ (fixed effects) = .07, *R* ^2^ (total) = .72 (see Table 1, Model 1).

**TABLE 1:** Linear mixed model testing main (Step 1) and quadratic (Step 2) associations of intergroup perceptions with cultural adoption as outcome in Study 1.

	*B*	95% CI		*t*	*df*	*p*
Model 1						
(Intercept)	1.16	0.90	1.42	8.84	30.20	<.001
Warmth	0.12	0.07	0.17	4.42	15.38	<.001
Competence	0.04	0.00	0.07	2.04	118.65	.043
Morality	0.11	0.06	0.15	4.58	21.68	<.001
Model 2						
(Intercept)	1.54	1.22	1.87	9.37	49.37	<.001
Warmth	0.01	−0.07	0.08	0.13	67.58	.896
Competence	−0.06	−0.12	0.01	−1.73	135.54	.086
Morality	0.10	0.06	0.15	4.39	27.05	<.001
Warmth × Competence	0.03	0.01	0.04	3.73	470.39	<.001

**FIGURE 2:**
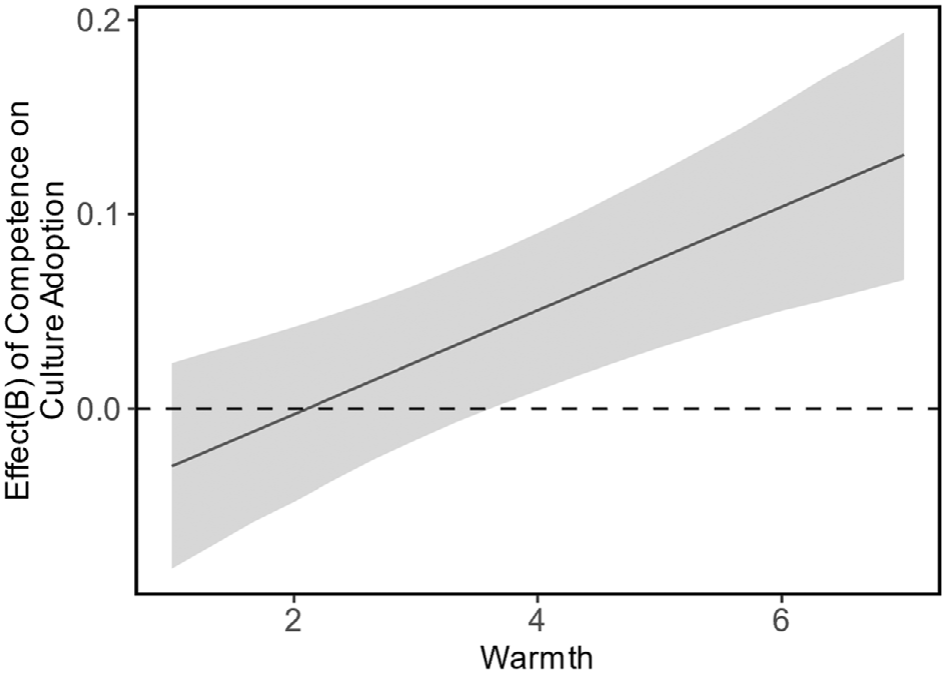
Association of competence with culture adoption at different levels of warmth in Study 1. Ribbons represent 95% confidence intervals.

Next, competence and warmth interacted significantly (see Table 1, Model 2), supporting H3, AIC = 6949.28, BIC = 7056.07, *R* ^2^ (fixed effects) = .07, *R* ^2^ (total) = .72. This model outperformed the the fit of the previous model, *χ* ^2^(1) = 12.20, *p* < .001. As displayed in Figure 2, the association between competence and culture adoption was non‐significant when warmth was low but became increasingly positive the warmer the target group was perceived to be. We explored interactions of morality with warmth (*p =* .685) and with competence (*p* = .070), but these effects did not reach statistical significance. In an extended model, the three‐way interaction between the three stereotype dimensions was also statistically non‐significant, *p* = .354.

#### Modelling interactions with in‐group perceptions

We then tested our two competing hypotheses. The first hypothesis predicted that culture adoption would be the highest when immigrants were scored higher than the in‐group on the three dimensions of intergroup perceptions (H5a). We tested this prediction using linear moderation analyses, yet none of the moderations reached significance, see Table 2, Model 1; AIC = 6957.62, BIC = 7093.95, *R* ^2^ (fixed effects) = .10, *R* ^2^ (total) = .73.

**TABLE 2:** Mixed model results testing whether the ratings of one's own group linearly (Model 1) or curvilinearly (Model 2) moderate the associations of the intergroup perception dimensions with culture adoption.

	*B*	95% CI		*t*	*df*	*p*
Model 1						
(Intercept)	0.15	−0.90	1.20	0.28	350.54	.779
Warmth	0.12	−0.01	0.24	1.84	423.58	.066
Competence	0.14	0.02	0.27	2.22	2342.41	.027
Morality	−0.01	−0.16	0.14	−0.11	1398.99	.915
Own Warmth	0.05	−0.16	0.26	0.43	280.26	.668
Own Competence	0.24	0.02	0.47	2.13	289.18	.030
Own Morality	−0.10	−0.35	0.16	−0.74	270.74	.460
Warmth × Own Warmth	0.00	−0.02	0.02	0.06	2604.25	.950
Competence × Own Competence	−0.02	−0.04	0.00	−1.73	2586.84	.080
Morality × Own Morality	0.02	−0.01	0.05	1.55	2607.75	.120
Model 2						
(Intercept)	2.22	2.04	2.40	24.04	158.60	<.001
Warmth Difference	10.80	6.42	15.17	4.84	27.32	<.001
Morality Difference	9.56	4.95	14.18	4.06	28.87	<.001
Competence Difference	2.73	−0.95	6.42	1.46	62.89	.150
Warmth Difference^2^	1.66	−0.81	4.13	1.32	685.44	.188
Morality Difference^2^	−1.29	−3.72	1.14	−1.04	888.40	.298
Competence Difference^2^	2.24	−0.09	4.58	1.89	1570.69	.059

The second competing hypothesis predicted that culture adoption would be the highest when a group is rated similarly to the in‐group (H5b). We tested this prediction by calculating difference scores between the own and out‐group ratings for each dimension and testing their curvilinear associations. Following this procedure, the highest level of culture adoption should be observed when the difference score is 0 (i.e., indicating that the in‐ and out‐groups are rated the same). No evidence for this prediction was found, AIC = 6920.37, BIC = 7038.91, *R* ^2^ (fixed effects) = .07, *R* ^2^ (total) = .75, see Table 2, Model 2 for the coefficients. The non‐significant results were also replicated when using piece‐wise regression instead of difference scores (see syntax in the Supporting Information).

#### Testing whether the associations between culture adoption and maintenance are moderated by intergroup perceptions

**FIGURE 3:**
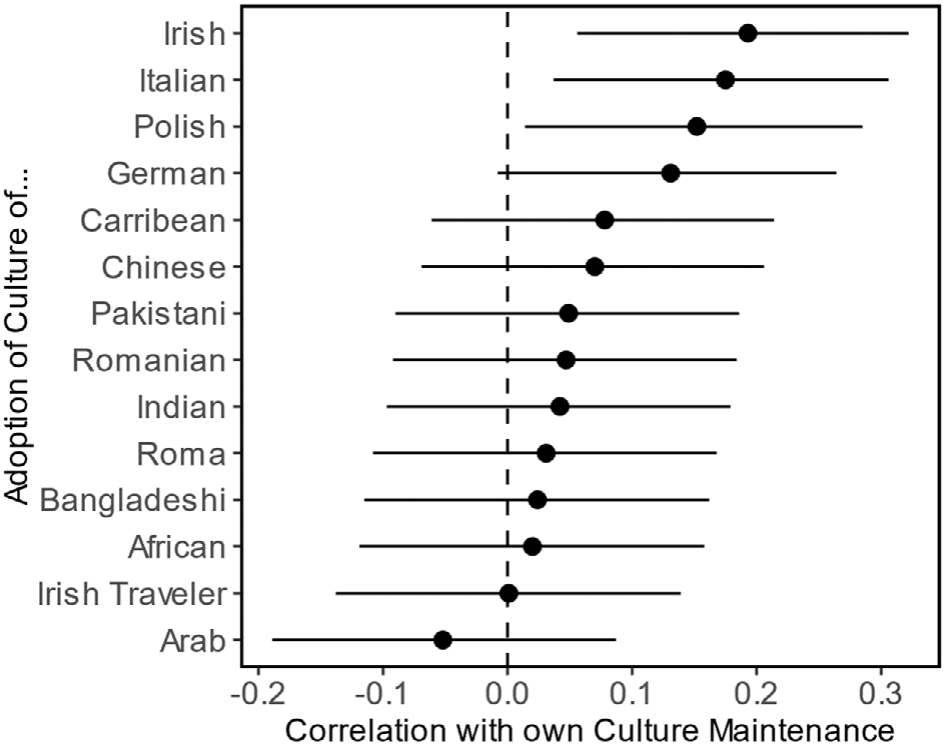
Correlations between own culture maintenance and adoption of the culture of various immigrant groups in Study 1. The point estimates reflect the Pearson correlation coefficients. Error bars represent 95% confidence intervals.

We estimated and visualized the correlation between participants' own culture maintenance and their adoption of the culture from each target group. As displayed in Figure 3, participants' own culture maintenance was positively associated with adopting Irish, Italian and Polish immigrants' culture and marginally with German immigrants' culture. However, the correlation was statistically non‐significant for the remaining target groups and, notably, trended negatively only for Arab immigrants.

**FIGURE 4:**
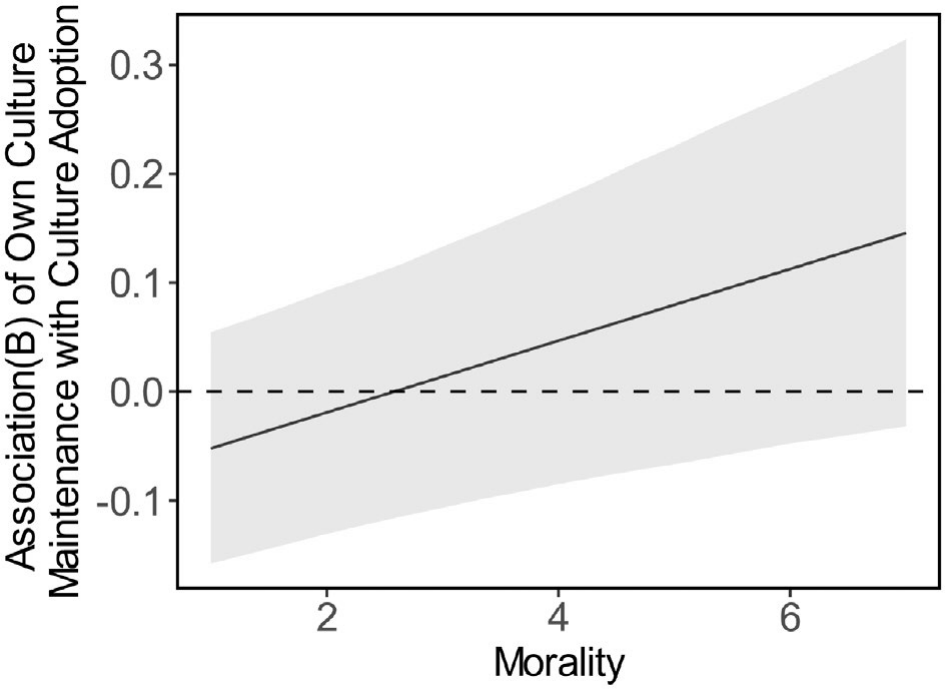
Association between own culture maintenance and other culture adoption at different levels of morality. Ribbons represent 95% confidence intervals.

We tested whether the dimensions of intergroup perception would moderate this association as predicted in H6, AIC = 6950.31, BIC = 7074.88, *R* ^2^ (fixed effects) = .09, *R* ^2^ (total) = .72. Indeed, morality statistically significantly moderated the relationship, but against expectations, not warmth and competence (see Table 3). As visualized in Figure 4, the association between own culture maintenance and other culture adoption became more positive the more moral the target group was perceived to be. However, the confidence intervals included zero at each level of the morality moderator, indicating that the results should be interpreted with caution.

**TABLE 3:** Linear mixed model results testing whether the association between own culture maintenance and other culture adoption was moderated by the intergroup perception dimensions in Study 1.

	*B*	95% CI		*t*	*df*	*p*
Intercept	1.57	1.04	2.10	5.76	268.76	<.001
Own culture maintenance	−0.09	−0.19	0.02	−1.62	347.42	.107
Warmth	0.07	−0.02	0.16	1.48	148.99	.142
Competence	0.07	−0.02	0.16	1.54	1349.82	.123
Morality	−0.05	−0.15	0.06	−0.84	512.06	.404
Own culture × warmth	0.01	−0.01	0.03	1.16	2453.54	.245
Own culture × competence	−0.01	−0.03	0.01	−0.86	2570.27	.391
Own culture × morality	0.03	0.01	0.05	3.10	2517.52	.002

### Preliminary discussion

As expected, the warmer, more competent and moral majority‐group members perceived immigrants to be, the more important they found it to adopt their culture. This pattern was observed at the individual and group levels, explaining why culture adoption was higher for immigrant groups that typically have higher status (e.g., Irish, German, Italian, Chinese^3^) than those with typically lower status (e.g., Gypsy or Irish Traveller, Roma, Romanian, Arab). As expected, the interaction between competence and warmth was significant, such that competence predicted higher levels of culture adoption only when groups were perceived as warm as well. This finding aligns well with the interactive nature of intergroup perceptions in producing social outcomes (Fiske, 2018; Fiske et al., 2002) and suggests that warmth offsets the potential threat that competent immigrant groups may elicit.

Against our predictions, we found no evidence for participants' evaluation of their own group moderating the associations of the three intergroup perception dimensions. Thus, whether immigrants and minority groups are perceived as similar or better than the in‐group does not seem to be associated with British majority‐group members' culture adoption.

The correlations between majority‐group members' culture maintenance and other culture adoption differed depending on the immigrant or minority group. This correlation was positive for groups from some Western European countries, whereas it became non‐significant for other groups. However, only the morality dimension seemed to explain some of these differences, tentatively suggesting that the relationship becomes more positive the more moral the out‐group is perceived to be.

This first study demonstrated that majority‐group acculturation differs depending on the type of immigrant and minority groups and the corresponding group perceptions. However, the study is limited by its cross‐sectional nature. Moreover, the three intergroup perception dimensions were relatively strongly positively associated (although factorially distinct), potentially making it statistically challenging to disentangle their unique effect. Therefore, in the following study, we aimed to replicate the results by separately manipulating the three intergroup perception dimensions in an experimental design. Moreover, we introduced perceptions of identity and economic indispensability (Mepham & Verkuyten, 2017; Verkuyten et al., 2014) as potential mediators.

## STUDY 2

As in Study 1, we hypothesized that the intergroup perception dimensions would predict higher levels of other culture adoption (H1) and expected a significant interaction between warmth and competence (H2). In addition, we introduced perceived economic and identity indispensability as potential mediators. We predicted that the three intergroup perception dimensions would directly inform perceptions of indispensability, with immigrant groups rated as warmer, more competent and moral being rated as more indispensable (H3). We explored whether the effects would differ depending on the type of indispensability in question. For instance, it could be that warmth plays more of a role in identity indispensability, whereas competence may play more of a role in economic indispensability for the reasons outlined in the main introduction. We further expected perceptions of indispensability to be associated with more other culture adoption (H4).

Unlike the first study, which assessed participants' ratings of actual immigrant groups, this and the following study experimentally manipulated perceptions of unspecified immigrant groups. While the use of real groups grounds the research in a naturalistic context with groups having established stereotypes and existing perceptions of indispensability, to understand the dynamics independent of pre‐existing perceptions of a particular group, as recommended by Esses et al. (1998) and Spencer‐Rodgers et al. (2007), Studies 2 and 3 used a complementary approach in which the immigrants group within the design was unspecified. This approach was adopted for several reasons. Practically, altering perceptions of real groups can be challenging because these perceptions are often deeply ingrained in people's experiences, ideological systems of thought and social structures. Methodologically, each real group is associated with pre‐existing stereotypes that vary widely. For example, one group might already be perceived as very warm, making it difficult to further increase this warmth compared to a group typically perceived as cold. Additionally, manipulations may have different effects depending on whether they align with or diverge from pre‐existing beliefs (Garcia‐Marques & Mackie, 1999; Hewstone, 1989). Modelling these complex processes is beyond the scope of the present study and does not further its immediate goals. Finally, ethically, it can be problematic to alter the stereotypes associated with real groups in a negative way, restricting the range of potential experimental manipulations. Therefore, using unspecified groups allowed for more controlled and generalized examination of perception changes. However, we return to the limitations of this approach in the general discussion section.

This study was conducted in the U.S., which has a long and complex history of immigration that has resulted in a highly diverse population. The statuses of various immigrant groups within American society differ substantially, influenced by factors such as the circumstances of their migration (voluntary or involuntary), their legal status and the historical and economic context in which they were received. Voluntary migrants seeking economic opportunities or educational advancement often experience different social receptions and are perceived fundamentally differently than to those fleeing political unrest, violence, or unlivable economic circumstances (Martinez et al., 2024). Documented immigrants generally find it easier to achieve higher social status compared to those without legal documentation, who may face significantly greater formal and informal barriers, including discrimination. Additionally, the context of reception, including public attitudes and government policies, can greatly impact the experiences of immigrant groups, with some communities being more welcomed and supported than others (Zhao & Biernat, 2022).

### Methods

#### Participants

A pre‐registered power simulation using the SIMR package (Green & MacLeod, 2016) suggested that we needed approximately 150 participants with each five responses (i.e., 750 trials) to achieve more than 90% power to detect a small to medium‐sized two‐way interaction (*d* = .25) at a .05 significance level. Thus, 150 participants from the U.S. were recruited via Prolific (*M*_age_ = 44.27; *SD*_age_ = 14.97). The sample was gender‐balanced (48.7% women, 50.8% men, 0.7% non‐binary/other) and close to politically representative of the U.S. according to polls at the time of data collection (30% Republicans, 30.7% Democrats, 38.7% Independents; cf. Gallup, 2023). As we were interested in the responses of White, non‐immigrant majority‐group members, two respondents who did not indicate their race and four who were born abroad were omitted from analyses, resulting in a final sample of 144 participants. None of them failed both attention checks, asking them to select a certain response (presented as part of two of the assessed measures). Thus, as pre‐registered, all participants were included in the analyses.

#### Procedure

The present study was pre‐registered at https://osf.io/pufs8/?view_only=dfed321a244841cb883f3075a5ef0bc2. Participants were told that they would be presented with information about how the U.S. population perceives five different immigrant groups in terms of three dimensions: competence, warmth and morality. They then completed five trials. In each trial, they saw bar charts with percentage estimates that indicated how U.S. Americans perceived the unnamed group of immigrants. The names of the immigrant groups were not specified for the reasons outlined in the introduction to this study. The bar chart contained the nine traits we assessed in the first study. Thus, each three corresponded to competence (i.e., intelligent, competent, skilled), morality (i.e., honest, sincere, trustworthy), and warmth (i.e., likable, friendly, warm). Notably, the estimates presented were randomized following a conjoint design. Specifically, the competence, morality and warmth clusters were separately assigned a random value from 5% to 95% for each trial. This value was then assigned to the three traits within the cluster with a random margin of ± 2% to create some variation, as would be the case in polls while ensuring that the trait ratings within each cluster were consistent. The bars were gradient coloured from red (0%) through yellow (50%) to green (100%). The three clusters were presented in random order. See Supporting Information for an example trial.

After reading how the U.S. population perceived the respective immigrant groups, participants were asked questions about the perceived identity indispensability (i.e., ‘To what extent do you think the immigrant group is indispensable for the cultural identity of the USA?’; 1 *not at all* – 5 *very much*) and economic indispensability (i.e., ‘To what extent do you think the immigrant group is indispensable for the economic functioning of the USA?’; 1 *not at all* – 5 *very much*) adopted from Fluit et al. (2023). Finally, they were asked to imagine that there were many immigrants from this group living in their neighbourhood and asked how they would relate to the group's culture on the 6‐item other culture adoption scale from the previous two studies (*α*s = .96–.97). Responses to these items were averaged so that higher scores represented more culture adoption. A CFA provided support for an oblique three‐factor solution in which the two indispensability dimensions were distinct from the cultural adoption dimension, *χ* ^2^(19) = 223.45, *p* < .001, CFI = 0.946, RMSEA = 0.123, SRMR = 0.038; but note that the RMSEA value was above the recommeded thresholds, which partly may be due to the relatively low *df* (Kenny et al., 2015).

#### Statistical analyses

The same analytic approach as in Study 1 was followed. Given the broad response scale for the independent variables (0–100 for the intergroup perception dimensions) relative to the other variables, these were standardized to achieve model convergence. Initially, we had pre‐registered to test for mediation using the mediation R package (Tingley et al., 2014), but we changed to multi‐level structural equation modelling in lavaan (Rosseel, 2012) after facing challenges with the mediation package due to lacking support for the data's multi‐level structure. We tested indirect effects using bootstrapped confidence intervals with 20,000 samples, a method recommended for multilevel mediation (Preacher & Selig, 2012).

### Results

#### Modelling the main and interactive experimental effects on culture adoption

We first tested whether warmth, competence and morality would lead to higher levels of other culture adoption (see Table 4). In the first model testing for the main effects, AIC = 2061.03, BIC = 2138.76, *R* ^2^ (fixed effects) = .11, *R* ^2^ (total) = .77, warmth and morality were associated with higher other culture adoption, whereas competence fell below the significance threshold, partly supporting H1. Unlike Study 1, and in contrast to H2, the interaction between warmth and competence was non‐significant in the second model, AIC = 2066.56, BIC = 2148.86, *R* ^2^ (fixed effects) = .11, *R* ^2^ (total) = .77. This model's fit was not significantly better than the model without interaction terms, *χ* ^2^(1) = 1.64, *p* = .200.

**TABLE 4:** Linear mixed model results testing main (Step 2) and quadratic (Step 2) effects on other cultural adoption in Study 2.

	*B*	95% CI		*t*	*df*	*p*
Model 1						
(Intercept)	3.23	3.01	3.45	28.53	105.99	<.001
Warmth	0.33	0.27	0.40	10.64	51.05	<.001
Competence	0.14	0.02	0.26	2.37	4.25	.073
Morality	0.38	0.30	0.45	10.20	6.77	<.001
Model 2						
(Intercept)	3.23	3.01	3.45	28.58	109.58	<.001
Warmth	0.34	0.28	0.40	10.73	54.61	<.001
Competence	0.14	0.03	0.25	2.42	4.25	.069
Morality	0.38	0.30	0.45	10.29	6.80	<.001
Warmth × Competence	0.04	−0.02	0.10	1.27	583.65	.205

We again explored interactions of morality with warmth (*B* = 0.10, *SE* = 0.03, *p* = .002) and competence (*p* = .337). An interactions plot showed that the effect of morality was positive at all levels of warmth but increased as the latter increased. The three‐way interaction between the stereotype dimensions was non‐significant in a further extended model (*p* = .379).

#### Modelling the main and interactive experimental effects on perceived indispensability

Next, we tested for the effects on perceived identity indispensability. Both indispensability perceptions were highly correlated, *r*(713) = .73, *p* < .001, in line with previous work (Fluit et al., 2023). Therefore, we first estimated effects across the two indispensability dimensions (added as additional level to the data) and then tested whether the type of indispensability moderated the effects. To achieve convergence, random slopes had to be dropped from these models. As seen in the first model in Table 5, AIC = 3769.75, BIC = 3811.87, *R* ^2^ (fixed effects) = .12, *R* ^2^ (total) = .59, each intergroup perception dimension positively predicted indispensability perceptions, supporting H3. In extended models, the two‐way and three‐way interactions between the dimensions had no statistically significant effect, *p*s > .080.

**TABLE 5:** Linear mixed model results testing main (Step 1) and moderated (Step 2) effects on indispensability in Study 2.

	*B*	95% CI		*t*	*df*	*p*
Model 1						
(Intercept)	3.10	2.94	3.26	38.19	79.89	<.001
Warmth	0.18	0.13	0.22	7.77	1326.27	<.001
Competence	0.27	0.22	0.32	11.44	1340.79	<.001
Morality	0.28	0.24	0.33	12.15	1329.00	<.001
Indispensability type^a^	−0.06	−0.14	0.02	−1.48	1278.89	.139
Model 2						
(Intercept)	3.10	2.94	3.26	38.19	79.88	<.001
Warmth	0.08	0.02	0.14	2.67	1303.04	.008
Competence	0.40	0.34	0.46	12.88	1310.89	<.001
Morality	0.25	0.19	0.31	8.29	1305.23	<.001
Indispensability type^a^	−0.06	−0.14	0.02	−1.52	1275.90	.130
Warmth × Type^a^	0.20	0.12	0.27	4.79	1275.90	<.001
Competence × Type^a^	−0.25	−0.33	−0.17	−6.20	1275.90	<.001
Morality × Type^a^	0.06	−0.02	0.14	1.42	1275.90	.155

^a^ Economic indispensability (1) vs. Identity indispensability (2).

**FIGURE 5:**
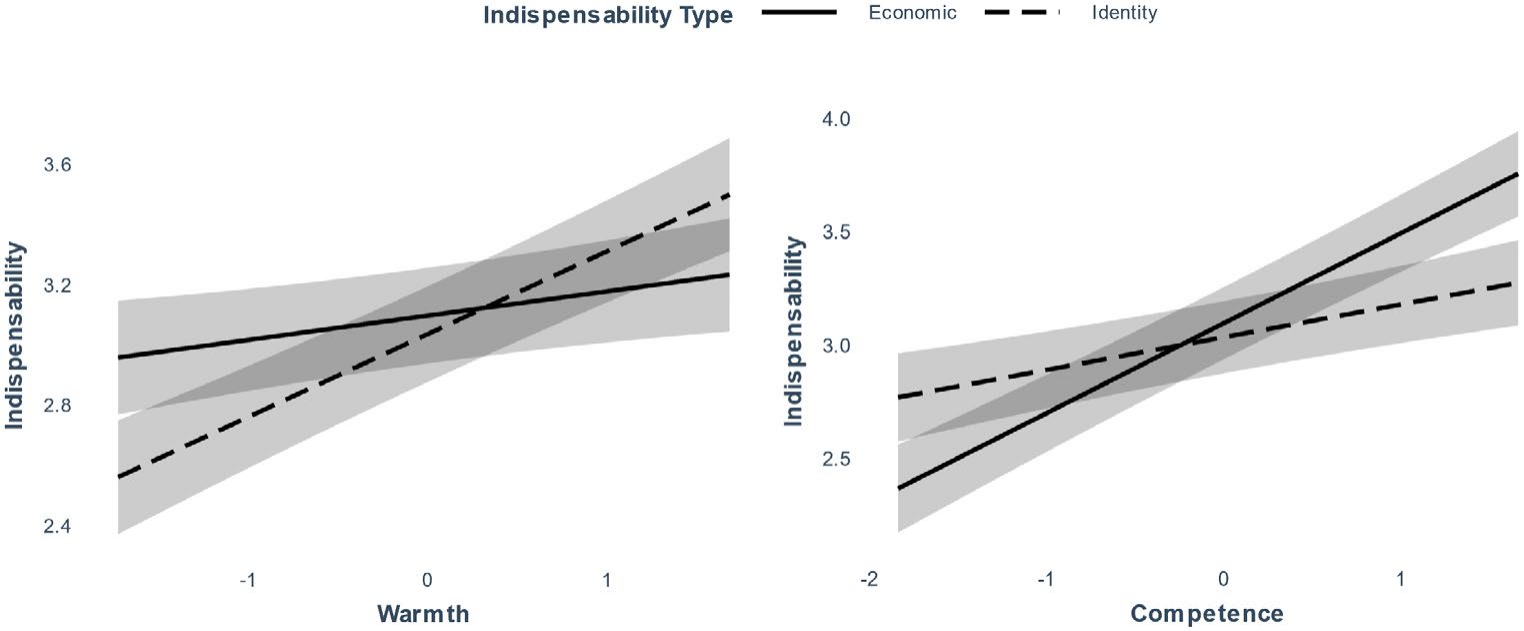
Effect of warmth and competence on economic and identity indispensability in Study 2. Ribbons represent 95% confidence intervals. The variables shown on the *x*‐axis are standardized.

However, in the next modelling step, AIC = 3729.14, BIC = 3787.06, *R* ^2^ (fixed effects) = .14, *R* ^2^ (total) = .61, the interactions between the indispensability type and warmth and between the indispensability type and competence were significant (see Table 5) and the model fit improved significantly, *χ* ^2^(3) = 60.50, *p* < .001. The effect of warmth was positive and significant for both types of indispensability but was stronger for identity indispensability, *B* = 0.28, *SE* = 0.03, *p* < .001, than for economic indispensability, *B* = 0.08, *SE* = 0.03, *p* = .008, see Figure 5. The opposite pattern was observed for competence, predicting economic indispensability more strongly, *B* = 0.40, *SE* = 0.03, *p* < .001, than identity indispensability, *B* = 0.14, *SE* = 0.03, *p* < .001.

#### Testing whether perceived indispensability mediates the effects

**FIGURE 6:**
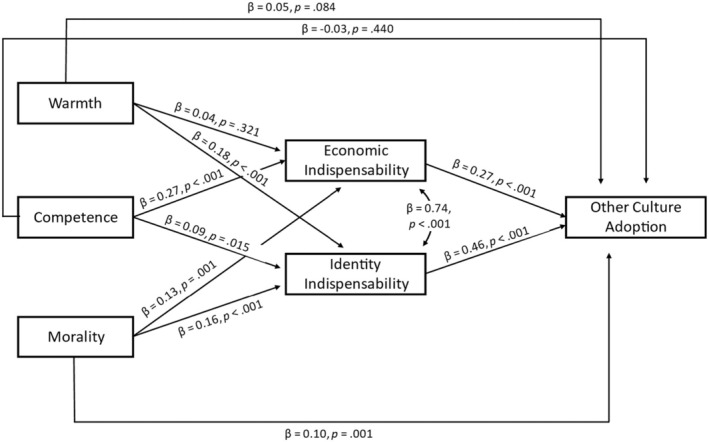
Mediation model tested in Study 2.

Given that two of three experimental factors predicted identity and economic indispensability differently, we estimated a multi‐level mediation model in which both indispensability variables (standardized in addition to the predictors) were considered as parallel mediators (see Figure 6). Please note that standard fit indices are not provided for multi‐level SEM models in lavaan. As can be seen, all effects of the intergroup perception dimensions on indispensability were positive and significant, except for the effect of warmth on economic indispensability. Both indispensability variables predicted more other culture adoption in line with H4 and their effects did not differ statistically according to a Wald's test, *p* = .300.

All indirect effects except for the effect of warmth going through economic indispensability reached significance (see Table 6). Wald's difference tests indicated that the indirect effect of warmth going through identity indispensability was significantly different from the corresponding indirect effect going through economic indispensability. For the other predictors, the indirect effects did not differ significantly.

**TABLE 6:** Indirect effects observed in Study 2.

Predictor/mediator	*β*	*B*	95% boot CI		*p* ^a^
			Lower	Upper	
Warmth					
Identity Indispensability	.08	0.005	0.002	0.008	<.001
Economic Indispensability	.01	0.001	−0.001	0.002	.331
Wald's Difference Test					< .001
Competence					
Identity Indispensability	.04	0.003	>0.001	0.005	.017
Economic Indispensability	.07	0.004	0.003	0.007	<.001
Wald's Difference Test					.128
Morality					
Identity Indispensability	.07	0.004	0.002	0.007	<.001
Economic Indispensability	.03	0.001	0.001	0.004	.008
Wald's Difference Test					.065

*Note*: Boot CI = Bootstrapped Intervals based on 20,000 samples.

^a^ *p*‐Value before applying Monte Carlo Standard Errors.

### Preliminary discussion

The second study experimentally replicated the findings from the first study. Warmth, competence and morality each independently made U.S. majority‐group members think it is more important to adopt the culture of immigrant groups. Critically extending the previous study, the effects of the intergroup perception dimensions were mediated by perceptions of indispensability. The more favourable immigrant groups were described to be in terms of each intergroup perception dimension, the more indispensable they were perceived to be for the U.S. identity and economy. However, some nuances emerged. Warmth primarily predicted identity indispensability, whereas competence primarily predicted economic indispensability. Morality seemed to predict both types of indispensability similarly. These findings make sense as competence can signal the immediate utility of immigrants for the labor market (Cuddy et al., 2011), whereas warmth may signal cooperativeness, approachability and prioritization of the group over personal interests (cf. Cislak & Wojciszke, 2008; Wyszynski et al., 2020). Morality may similarly predict economic and identity indispensability due to its relevance for both domains (Ellemers et al., 2017; Weiss et al., 2021).

Unlike Study 1, we found no significant interaction between warmth and competence. The interplay of warmth and competence may be more pronounced in naturalistic settings (i.e., as with natural immigrant groups in Study 1) than when the effect of both factors is isolated in more internally but less ecologically valid ways.

## STUDY 3

As the mediation in Study 2 was based on one causal path (intergroup perception on key dimensions → indispensability) and one correlational path (indispensability → culture adoption), we aimed to obtain evidence for the causal role of indispensability by manipulating it (Spencer et al., 2005). We presented a sample of U.S. participants with five unnamed immigrant groups. In each trial, they were informed how experts evaluated the respective immigrant group regarding its indispensability to the U.S. identity and economy. We then measured the degree to which the participants found it important to adopt the culture of the group. We expected both types of indispensability to have positive effects (H1) but tested whether the impact of identity indispensability would be more pronounced, as the trend suggested in Study 2 (H2). We also tested the prediction that culture adoption would be most pronounced when both types of indispensability are high (H3). Finally, we conducted a pre‐registered exploration of whether indispensability moderates the relationship between own culture maintenance and other culture adoption.

### Methods

#### Participants

Following the power simulation from Study 2, we collected 150 U.S. participants (*M*_age_ = 39.99; *SD*_age_ = 14.39) via Prolific who each provided five responses (i.e., 750 trials). Participants from the previous study, also conducted in the U.S., were prevented from participating in this study. The sample was gender‐balanced (48.0% women, 50.0% men, 2% non‐binary/other) and close to politically representative at the time of data collection (32% Republicans, 30.7% Democrats, 36.0% Independents, 1.3% other; cf. Gallup, 2023). All but one participant identified as White and all but three participants were born in the U.S. Due to our focus on White, non‐immigrant majority‐group members, these four responses were excluded from analyses, resulting in a sample of 146.

#### Procedure

The present study was pre‐registered at https://osf.io/5a4cb/?view_only=f5733a2a48d8479080d48bfcfd978051. Participants completed five trials. In each trial, they saw two bar charts with percentage estimates that indicated how a group of experts perceived an unnamed group of immigrants in terms of identity and economic indispensability (please see Supporting Information for the exact wording and instructions to participants). Both dimensions were independently randomized for each trial from 0 to 100% (indispensable) and coloured as in Study 2. The order of the two indispensability dimensions was randomized at the participant level. Having read the description, the participants indicated to what extent they found it important to adopt the group's culture on the same scale as used in the previous studies, with higher averaged scores meaning more culture adoption (*α*s = .93–.93). After all trials, they were asked to what extent they found it important to maintain their own mainstream culture (*α* = .94) on the same scales as in Study 1. Again, items were averaged so that higher scores indicated more culture maintenance.

### Results

#### Testing for the effects of perceived indispensability on cultural adoption

To achieve model convergence, we standardized the two experimental predictor variables. Testing the first hypothesis, we estimated a model in which the two indispensability variables had main effects, AIC = 2011.87, BIC = 2066.99, *R* ^2^ (fixed effects) = .07, *R* ^2^ (total) = .74. The intercepts and both slopes were set to random. Both indispensability conditions similarly predicted higher levels of other culture adoption (see Table 7, Model 1) and did not differ significantly when type of indispensability was added as a moderating factor, *B* = .02, *SE* = .04, *t*(1319) = 0.41, *p* = .683. Thus, H1 but not H2 was confirmed. In the second model, against H3, the interaction between both factors was non‐significant, AIC = 2017.16, BIC = 2076.87, *R* ^2^ (fixed effects) = .07, *R* ^2^ (total) = .74 (see Table 7, Model 2). The second model did not have significantly better model fit than the first model, *χ* ^2^(1) = 1.87, *p* = .172.

**TABLE 7:** Linear mixed model results testing main (Step 1) and moderated (Step 2) effects on other culture adoption in Study 3.

	*B*	95% CI		*t*	*df*	*p*
Model 1						
(Intercept)	3.43	3.24	3.63	34.58	144.47	<.001
Identity Indispensability	0.28	0.21	0.36	7.54	3.91	.002
Economic Indispensability	0.26	0.21	0.32	8.89	473.84	<.001
Model 2						
(Intercept)	3.43	3.24	3.63	34.56	144.98	<.001
Identity Indispensability	0.28	0.21	0.36	7.36	3.90	.002
Economic Indispensability	0.27	0.21	0.32	8.94	556.26	<.001
Identity × Economic	−0.04	−0.10	0.02	−1.38	600.86	.167
Model 3						
Intercept	2.75	2.23	3.26	10.45	143.99	<.001
Own Culture Maintenance	0.18	0.05	0.30	2.83	143.98	.005
Identity Indispensability	−0.06	−0.22	0.10	−0.72	97.66	.477
Economic Indispensability	0.07	−0.08	0.22	0.94	552.53	.347
Maintenance × Identity	0.09	0.05	0.13	4.69	597.61	<.001
Maintenance × Economic	0.05	0.02	0.09	2.80	597.79	.005

#### Testing whether perceived indispensability moderates association between culture maintenance and culture adoption

**FIGURE 7:**
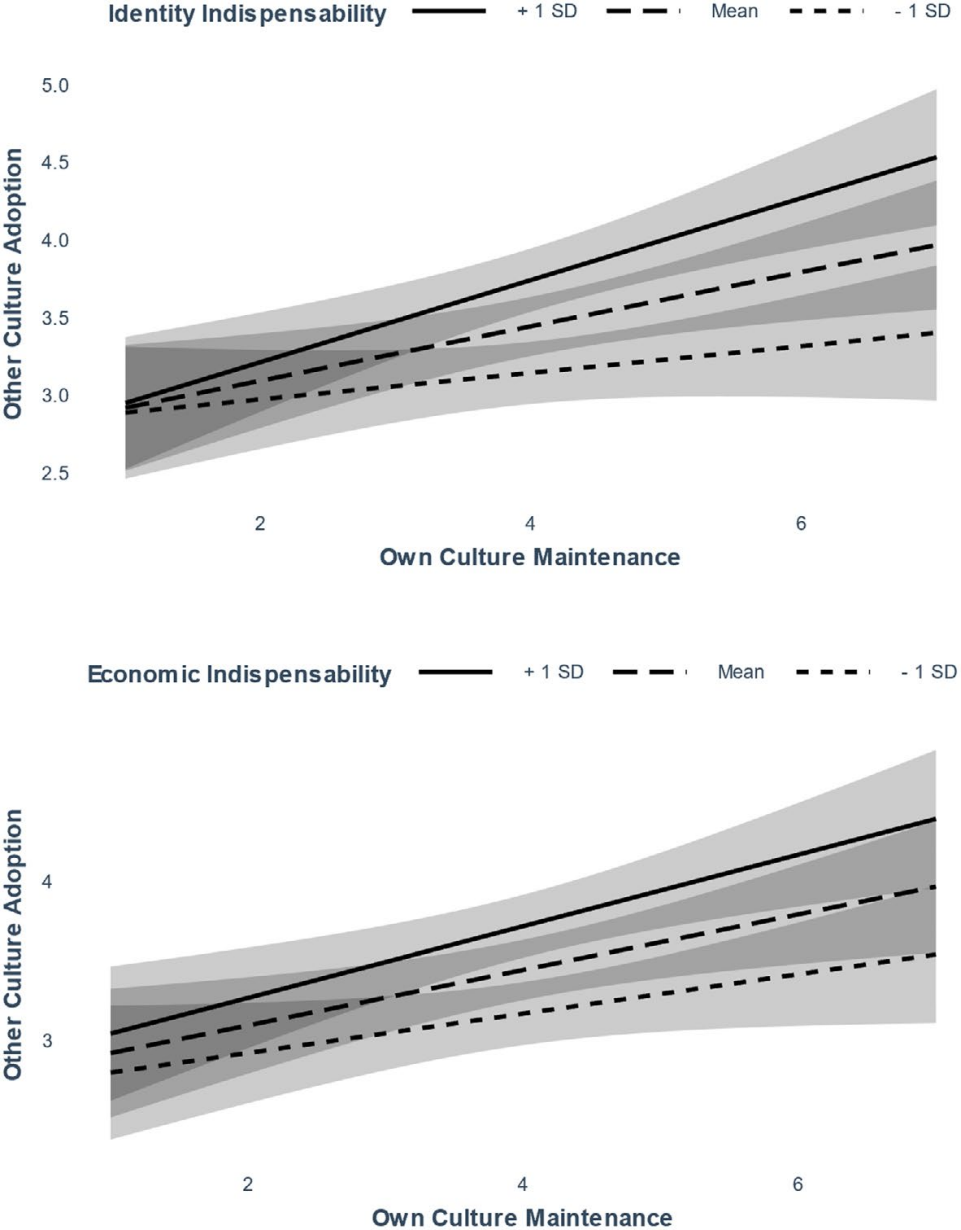
Association between own culture and other culture maintenance as moderated by the indispensability manipulations in Study 3. Ribbons represent 95% confidence intervals.

Next, as pre‐registered, we explored whether economic and identity indispensability would moderate the association between own culture maintenance and other culture adoption, AIC = 1995.73, BIC = 2064.62, *R* ^2^ (fixed effects) = .12, *R* ^2^ (total) = .75, see Table 7, Model 3. Indeed, both interactions were highly significant. As presented in Figure 7, at moderate, *B* = 0.17, *SE* = 0.06, *p* = .005, and especially at high levels of identity indispensability, *B* = 0.26, *SE* = 0.06, *p* < .001, own culture maintenance and other culture adoption were statistically significantly and positively associated, but not at low levels of identity indispensability, *B =* 0.09, *SE* = 0.06, *p* = .187. Similarly, own culture maintenance and other culture adoption were statistically significantly and positively associated at moderate, *B =* 0.17, *SE* = 0.06, *p* = .005 and especially at high levels of economic indispensability, *B =* 0.23, *SE* = 0.06, *p* < .001, but not at low levels of economic indispensability, *B =* 0.12, *SE* = 0.06, *p* = .055.

### Preliminary discussion

The third study provided further causal support for the mediational model. Manipulating identity and economic indispensability made majority‐group members more willing to adopt the culture of immigrants. Moreover, when indispensability was moderate or high, participants' preference for own culture maintenance became positively associated with their motivation to adopt the culture of immigrants. In other words, the more indispensable immigrants were described to be to the U.S. identity or economy, the more the two acculturation orientations became reconcilable.

## GENERAL DISCUSSION

Majority‐group acculturation has received growing research attention over the recent years (Kunst, Lefringhausen, Sam, et al., 2021). However, most existing research is limited in that it has treated immigrant and minority‐group cultures as one homogeneous entity. In one correlational and two experimental studies, we therefore investigated how the ways different immigrant and minority groups are perceived can influence majority‐group members' acculturation in systematic ways.

In the first study, when British majority‐group members perceived immigrant and minority groups as warm, competent and moral, they were more inclined to adopt their culture. The second study experimentally replicated these findings in the U.S. and demonstrated that perceptions of indispensability correlationally mediated the effects of the key intergroup perception dimensions on majority‐group acculturation. In the third study, we provided additional support for the mediational model, demonstrating the causal effect of indispensability on majority‐group members' adoption of the culture of immigrants.

Together, our studies emphasize the need for majority‐group acculturation research to look beyond the adoption or rejection of the culture of immigrants or minority groups generically if it aims at mapping out the complexities of these processes. Almost all existing studies on the topic have investigated majority‐group members' orientation towards ‘immigrants’ or ‘minority groups’ generally (Kunst, Lefringhausen, Sam, et al., 2021). While there are legitimate contexts in which scholars might be interested in these generalized attitudes towards overarching categories, our findings reveal that majority‐group members' acculturation processes vary significantly when it comes to more specific subgroups. These differences are largely driven by perceived distinctions between the groups.

The present set of studies suggests that the three assessed dimensions of intergroup perceptions predominantly exert independent effects on the culture adoption of majority‐group members. Evidence for interactions among the dimensions was limited. Notably, only in the initial study did the dimensions of warmth and competence exhibit the hypothesized interaction (Fiske et al., 2002), with the particular combination of high warmth and high competence showing a distinctively high level of willingness to adopt the cultures of immigrants. Conversely, in the second study, the influence of warmth on cultural adoption was amplified with an increase in the perception of morality. The results from our second experiment suggest that warmth and morality, when manipulated independently, exert synergistic effects. The perception of a group of immigrants as warm, suggesting cooperativeness and a prioritization of group interests over individual ones (Cislak & Wojciszke, 2008), coupled with a perception of morality, potentially indicating trustworthiness (Weiss et al., 2021), appears to enhance the willingness of majority‐group members to adopt the cultural practices of otherwise unspecified immigrant groups. However, the absence of such an interaction (i.e., warmth × morality) in the more ecologically valid setting of Study 1, where the three dimensions were highly interrelated, necessitates further replication of this finding. Moreover, it should be noted that the distinction between morality and warmth as separate dimensions, or the consideration of morality as a facet of warmth, remains a subject of debate (Cuddy et al., 2011; Leach et al., 2007).

The current research contributes to and expands upon existing literature (Guerra et al., 2015; Mepham & Verkuyten, 2017; Verkuyten et al., 2014; Verkuyten & Khan, 2012), highlighting the potential significance of perceptions of indispensability in shaping not only intercultural relations but also acculturation orientations. Furthermore, it elucidates the connection between perceptions of groups along key dimensions and the cultural orientations individuals adopt towards these groups. Specifically, perceptions of warmth, competence and morality initially seem to determine the extent to which immigrants are deemed to make essential contributions upon which society may rely. When such perceptions of indispensability are elevated, majority‐group members are arguably more likely to regard these immigrants as qualified prospective members of their society, from whom it is beneficial to learn and with whom social association is deemed valuable. Future research should explore the underlying mechanisms of this linkage and its potential adaptiveness (see Kunst & Mesoudi, 2024). Adopting the culture of a group perceived as indispensable to the societal group identity may serve to enhance one's collective self‐esteem through the acquisition of the positively valued traits that facilitated this cultural adoption in the first place. By adopting the culture of economically indispensable groups, individuals may aim to acquire traits that enhance their group's and/or own economic prospects within society.

Our findings hold significant repercussions for the evolving dynamics of cultural shifts and intercultural interactions over the course of time. It appears that the cultural influence of those immigrant and minority groups that are viewed as less desirable based on key dimensions of intergroup perception and perceived contributions may be limited in shaping the majority culture. It is crucial to acknowledge that these intergroup perceptions and perceptions of indispensability largely mirror historically entrenched and systemic biases due to inequalities (Caprariello et al., 2009; Haslam et al., 2002) rather than the factual attributes of the groups. Consequently, these intergroup perceptions and the ensuing perceived indispensability can drive majority‐group individuals to dismiss the culture of groups already marginalized within society. On a collective scale, this process may inhibit cultural convergence, thereby sustaining intercultural divides (Byrne, 1997; Montoya et al., 2008). Historically, the processes delineated in our research could explain transcultural dynamics, leading to the amalgamation of certain (higher status) groups, while others remain marginalized. Such processes have significant implications for the extent to which members of low‐status and stigmatized groups (for example, asylum seekers, refugees and people of a different religion than the majority) are permitted to contribute to the cultural development of their societies of residence.

At the same time, our research underscores the importance of countering negative intergroup perceptions by framing the characterizations of immigrants around their inherent strengths instead of their perceived weaknesses. This approach may enhance the cultural influence of immigrants, making members of the majority group more receptive to adopting elements from immigrant cultures. Looking ahead, it is essential for future studies to explore actionable, scalable strategies that can be put into place to cultivate these positive dynamics.

Whereas our findings are suggestive, they should be interpreted in light of several constraints on generality (Simons et al., 2017). First, our studies were conducted in two Western settings and with non‐random samples. Thus, although the samples were gender‐balanced and politically close to representative, future research is needed to establish the generalizability of our findings, especially in non‐Western contexts.

The use of single items to measure the indispensability of immigrants in Study 2 may also have limitations. Although the two items were adopted from previous research (Fluit et al., 2023) and often showed distinct associations with the other variables, the items correlated highly and it can be debated whether single‐item scales as compared to multi‐item scales are optimal (Bergkvist & Rossiter, 2007; Diamantopoulos et al., 2012). Although the converging results from the correlational measurement and experimental manipulation of indispensability give us some confidence in the results, future research may profitably use multi‐item indispensability scales, such as the one developed by Guerra et al. (2016).

Although we find evidence for key intergroup perceptions influencing perceptions of indispensability, the opposite direction of causality is also plausible. If a group is described as indispensable, this may elicit expectations of specific group attributes, including intergroup perceptions of warmth, competence and morality (see Durante et al., 2010). Future research could test these possibly reciprocal processes. Furthermore, subsequent research could incorporate manipulation checks to assess the specificity of the experimental manipulations. Such checks could examine, for example, the extent to which altering one construct (e.g., warmth, economic indispensability) influences another (e.g., competence, identity indispensability, correspondingly). We would assume these constructs to be interconnected via halo effects, representing an intrinsic challenge in the experimental manipulation of variables generally correlated in naturalistic contexts.

Furthermore, we elected to manipulate the dimensions of social evaluations (Study 2) and identity indispensability (Study 3) by presenting participants with evaluations of groups of immigrants as conducted by either the general population or a select group of experts, respectively. Our consistent findings of effects suggest that this manipulation predictably influenced participants' evaluations of an otherwise unspecified group (that is, this information formed the sole basis of their judgement in the absence of pre‐existing beliefs). However, the extent to which the manipulation of each manipulation altered perceptions of the groups may differ and warrants direct examination.

We also acknowledge that using unspecified groups, while increasing the internal validity of our research, decontextualizes the immigrant groups in question. Incorporating intersecting factors such as the status of immigrant groups, their voluntariness and their racialization could add important nuance in future work (Esses, 2021). This approach would allow for a more detailed understanding of how these variables influence perceptions and improve the contextual relevance and applicability of the findings. It is also vital to note that the delineation of groups' warmth, competence and morality in the latter two studies could potentially have primed participants to envision particular immigrant groups that align with these descriptions in their perceptions. In future research, the influence of such pre‐existing biases could be limited by using fictional immigrant group names (Esses et al., 1998), although these may also make the design less credible.

Participants' acculturation orientations were assessed using the conventional methodology prevalent in the field. However, a significant limitation inherent to this widely adopted approach is its failure to account for the extent of participants' actual knowledge about other cultures. Consequently, while acculturation orientations were evaluated across a range of standard domains, including tradition, values and identity, the precise manner in which participants mentally represented the content within these domains remains unclear. Future research could therefore benefit from efforts to replicate our findings by employing qualitative methods that incorporate open‐ended questions, prompting participants to explicitly detail the specific aspects they adopt from other cultures. Alternatively, quantitative approaches could be devised to rigorously assess participants' knowledge of these cultural aspects.

For readers who are mwell‐versed in the literature pertaining to intergroup contact than acculturation, the inclusion of a contact domain in the assessment of cultural adoption might appear unconventional. Nonetheless, it is important to recognize that intergroup contact has been a fundamental aspect of acculturation orientations since the inception of Berry's (1997) influential theoretical framework. This dimension is a conventional component that acculturation orientation scales include. Moreover, in our research, the item related to contact exhibited consistent factorial alignment with the other cultural adoption items.

While not a focal part of our paper, we also explored whether SDO moderated the effects in the first two studies (detailed results are reported in the Supporting Information). In Study 1, only one moderation with SDO reached significance and this moderation was curvilinear. Specifically, majority‐group members high in SDO showed little culture adoption from groups they perceived as low in competence, which is in line with work showing that those high in SDO typically dislike low‐status out‐groups (Duckitt & Sibley, 2007). However, interestingly, they also seemed to adopt the culture of immigrant groups less when these groups were very high in competence, arguably because these groups were seen as a threat to the social hierarchy (Esses et al., 1998). However, this association should be interpreted cautiously, as it did not experimentally replicate in Study 2. One reason for this could be the decontextualized nature of using unspecified groups – a limitation discussed previously.

Some of our analyses focused on majority‐group members' culture maintenance. A point of criticism could be the phrasing of our measure for this purpose, which assessed the maintenance of the ‘British mainstream’ culture. This term was deliberately chosen to highlight the culture of the majority group, specifically White Britons, who constituted our participant pool. However, as noted in our introduction, the British mainstream culture has continuously been shaped by cultural elements introduced by immigrants at various points of history. Consequently, the precise understanding of this culture may vary among individuals, influenced by their ethnic or civic perceptions of nationhood (Leong et al., 2020). Future research should consider providing a clear definition of this mainstream culture to participants. Alternatively, it may adopt a more specific term such as ‘White British mainstream culture’ in its methodology. Broader and more inclusive definitions may generally allow for more groups to be viewed as prototypical and indispensable (Waldzus et al., 2003). It would thus be interesting to test the degree to which participants had narrow or complex definitions of the mainstream culture as a moderating factor. More complex and permeable (e.g., civic) conceptualizations of the mainstream culture may generally increase the effect of intergroup perceptions of warmth, competence and morality on indispensability beliefs and acculturation orientations, whereas narrow (e.g., ethnic) conceptualizations may restrict the influence of these perceptions.

Finally, it is important to note that intergroup perceptions can be conceptualized in many ways. For instance, a three‐factorial model of intergroup perceptions that distinguishes between the factors of agency/socioeconomic success, conservative‐progressive beliefs and communion is well supported by bottom‐up, data‐driven studies (Koch et al., 2016).

## CONCLUSION

The present research emphasizes the need for research on the acculturation of majority groups to move beyond the adoption of the culture of ‘immigrants’ or ‘minority groups’ broadly. Our results demonstrate that majority‐group members are motivated to adopt the culture of immigrants differently based on how they perceive them in terms of warmth, competence, morality and subsequently indispensability for the mainstream society's identity and economic functioning. This selective cultural adoption has important consequences for cultural dynamics and social equality in evolving diverse societies.

## AUTHOR CONTRIBUTIONS

**Jonas R. Kunst:** Conceptualization; investigation; writing – original draft; writing – review and editing; visualization; validation; methodology; formal analysis; project administration; data curation. **Kinga Bierwiaczonek:** Investigation; writing – review and editing; methodology; formal analysis. **Milan Obaidi:** Conceptualization; writing – review and editing. **Sam Fluit:** Conceptualization; writing – review and editing. **Tilmann von Soest:** Conceptualization; writing – review and editing. **David Sam:** Conceptualization; writing – review and editing. **John F. Dovidio:** Conceptualization; investigation; writing – original draft; writing – review and editing.

## CONFLICT OF INTEREST STATEMENT

The authors confirm that they carried out the research without any commercial or financial ties that could be interpreted as potential conflicts of interest.

## Supporting information


Appendix S1.

## Data Availability

The data that support the findings of this study are openly available in OSF at https://osf.io/gav7r/?view_only=cd41212909444228b5e345426df61ead.

## Footnotes

^1^
For this and the second study, we also explored potential curvilinearity and the moderating role of social dominance orientation (SDO). Due to space limitations, these results are presented in the Supporting Information, and the SDO results are summarized briefly in the general discussion.

^2^
The power analysis was conducted to assess the feasibility of a cross‐level interaction in exploratory analyses involving SDO, presented in Supporting Information. This approach was selected because it requires a significantly larger sample size than that needed for trial‐level interactions, presented in the main text.

^3^
This assessment of status was based on relatively high ratings of Chinese immigrants in the first study in conjunction with cross‐cultural work on stereotypes associated with ‘Asians’ (Cuddy et al., 2009), particularly on dimensions such as competence, which is closely related to perceptions of social status, as well as morality and warmth. However, we acknowledge that in many Western contexts, Chinese immigrants are devalued by the majority society and experience high levels of discrimination (often *because of* their high perceived status and perceptions of threat).
