# Supplementary material for: Selective cultural adoption: The roles of warmth, competence, morality and perceived indispensability in majority‐group acculturation
Source: Br J Soc Psychol. 2024 Sep 16;64(2):e12801. doi: 10.1111/bjso.12801 (PMC11923941; doi:10.1111/bjso.12801)
Supplement: Supplementary file 1 — Appendix S1. [file BJSO-64-0-s001.pdf]

Supplementary Online Materials

Contents

Table S1 *Participant Demographics in Study 1* 2

Table S2 *Correlations Between Variables at the Trial Level in Study 1* 4

Table S3 *Correlations Between Variables at the Participant Level in Study 1* 5

Table S4 *Correlations Between Variables at the Trial Level in Study 2* 6

Table S5 *Correlations Between Variables at the Participant Level in Study 2* 7

Figure S1. *The Interaction between Morality and Warmth in Study 2* 8

Table S6. *Correlations Between Variables at the Trial Level in Study 3* 9

Study 1: SDO Analysis 10

Table S7. *Linear Mixed Model Results Testing Whether the Association Between the Intergroup Perception Dimensions and Other Culture Adoption was Moderated by SDO in Study 1* 11

Figure S2. *Curvilinear Effect of Competence on Culture Adoption at Low, Medium, and High Levels of SDO in Study 1* 12

Study 2: SDO Analysis 12

Tests of Curvilinearity 13

Study 1: Test of Quadratic Effects 13

Table S8 *Linear Mixed Model Results Testing Main (Step 1) and Quadratic (Step 2) Associations with Cultural Adoption in Study 1* 13

Study 2: Test of Quadratic Effects 14

Figure S4. *Quadratic Relationship Between Competence and Other Culture Adoption in Study 2* 15

Table S1 *Participant Demographics in Study 1*

Variable		%
Inhabitants at place of living	More than 1 million	15.4
	300,000 - 1 million	15.9
	100,000 - 300,000	16.9
	50,000 - 100,000	14.4
	10,000 - 50,000	16.4
	5,000 - 10,000	10.4
	Less than 5000	10.4
Education	Secondary education	10.4
	High school diploma	11.9
	Technical/community college	17.9
	Undergraduate degree (BA/BSc/other)	34.3
	Graduate degree (MA/MSc/MPhil/other)	23.4
	Doctorate degree (PhD/other)	2.0
Occupation	Student	3.5
	Employed full-time	58.7
	Employed part-time	15.4
	Unemployed (and job seeking)	3.5
	Sick leave/disability benefit	2.0
	Not in paid work (e.g., homemaker, retired)	14.4
	Other	2.5
Income	Less than $10.000[a]	11.9
	$10.000 - $19.999	15.4
	$20.000 - $29.999	20.9
	$30.000 - $39.999	15.9
	$40.000 - $49.999	9.0
	$50.000 - $59.999	10.9
	More than $60.000	8.0
	Rather not say	8.0
Political Affiliation	Labour	47.5
	Conservatives	22.2
	Liberal Democrats	9.1

Reform	3.5
Greens	4.0
SNP	3.5
UKIP	0.5
Other	9.6

Note. [a]Please note that income was assessed as USD rather than GBP due to an oversight when designing the survey.

Table S2 *Correlations Between Variables at the Trial Level in Study 1*

		warmth	competence	morality	adoption
warmth	Pearson Correlation	1	,633**	,776**	,318**
	Sig. (2-tailed)		<,001	<,001	<,001
	N	2809	2799	2796	2809
competence	Pearson Correlation	,633**	1	,794**	,316**
	Sig. (2-tailed)	<,001		<,001	<,001
	N	2799	2802	2789	2802
morality	Pearson Correlation	,776**	,794**	1	,361**
	Sig. (2-tailed)	<,001	<,001		<,001
	N	2796	2789	2799	2799
adoption	Pearson Correlation	,318**	,316**	,361**	1
	Sig. (2-tailed)	<,001	<,001	<,001	
	N	2809	2802	2799	2814

**. Correlation is significant at the 0.01 level (2-tailed).

Table S3 *Correlations Between Variables at the Participant Level in Study 1*

		SDO_MEAN_mean	warmth_own_mean	competence_own_mean	morality_own_mean	warmth_mean	competence_mean	morality_mean	own_maintenance_mean	adoption_mean
SDO_MEAN_mean	Pearson Correlation	1	-,139*	-,093	-,128	-,173*	-,236**	-,198**	,296**	-,203**
	Sig. (2-tailed)		,050	,190	,072	,014	<,001	,005	<,001	,004
	N	201	201	201	200	201	201	201	201	201
warmth_own_mean	Pearson Correlation	-,139*	1	,527**	,690**	,171*	,196**	,169*	,068	,145*
	Sig. (2-tailed)	,050		<,001	<,001	,015	,005	,017	,337	,040
	N	201	201	201	200	201	201	201	201	201
competence_own_mean	Pearson Correlation	-,093	,527**	1	,672**	,074	,144*	,100	,082	,175*
	Sig. (2-tailed)	,190	<,001		<,001	,295	,042	,156	,249	,013
	N	201	201	201	200	201	201	201	201	201
morality_own_mean	Pearson Correlation	-,128	,690**	,672**	1	,198**	,197**	,238**	,047	,157*
	Sig. (2-tailed)	,072	<,001	<,001		,005	,005	<,001	,508	,026
	N	200	200	200	200	200	200	200	200	200
warmth_mean	Pearson Correlation	-,173*	,171*	,074	,198**	1	,856**	,901**	,065	,295**
	Sig. (2-tailed)	,014	,015	,295	,005		<,001	<,001	,360	<,001
	N	201	201	201	200	201	201	201	201	201
competence_mean	Pearson Correlation	-,236**	,196**	,144*	,197**	,856**	1	,899**	,144*	,374**
	Sig. (2-tailed)	<,001	,005	,042	,005	<,001		<,001	,042	<,001
	N	201	201	201	200	201	201	201	201	201
morality_mean	Pearson Correlation	-,198**	,169*	,100	,238**	,901**	,899**	1	,100	,387**
	Sig. (2-tailed)	,005	,017	,156	<,001	<,001	<,001		,158	<,001
	N	201	201	201	200	201	201	201	201	201
own_maintenance_mean	Pearson Correlation	,296**	,068	,082	,047	,065	,144*	,100	1	,086
	Sig. (2-tailed)	<,001	,337	,249	,508	,360	,042	,158		,226
	N	201	201	201	200	201	201	201	201	201
adoption_mean	Pearson Correlation	-,203**	,145*	,175*	,157*	,295**	,374**	,387**	,086	1
	Sig. (2-tailed)	,004	,040	,013	,026	<,001	<,001	<,001	,226	
	N	201	201	201	200	201	201	201	201	201

*. Correlation is significant at the 0.05 level (2-tailed).

**. Correlation is significant at the 0.01 level (2-tailed).

Study 2

Table S4 *Correlations Between Variables at the Trial Level in Study 2*

		warmth	competence	morality	cultural indispensability	economic indispensability	cultural adoption
warmth	Pearson Correlation	1	,028	-,014	,215**	,059	,196**
	Sig. (2-tailed)		,455	,701	<,001	,113	<,001
	N	715	715	715	715	715	715
competence	Pearson Correlation	,028	1	,002	,104**	,307**	,063
	Sig. (2-tailed)	,455		,963	,005	<,001	,092
	N	715	715	715	715	715	715
morality	Pearson Correlation	-,014	,002	1	,208**	,161**	,247**
	Sig. (2-tailed)	,701	,963		<,001	<,001	<,001
	N	715	715	715	715	715	715
cultural indispensability	Pearson Correlation	,215**	,104**	,208**	1	,735**	,675**
	Sig. (2-tailed)	<,001	,005	<,001		<,001	<,001
	N	715	715	715	715	715	715
economic indispensability	Pearson Correlation	,059	,307**	,161**	,735**	1	,612**
	Sig. (2-tailed)	,113	<,001	<,001	<,001		<,001
	N	715	715	715	715	715	715
adoption	Pearson Correlation	,196**	,063	,247**	,675**	,612**	1
	Sig. (2-tailed)	<,001	,092	<,001	<,001	<,001	
	N	715	715	715	715	715	715

**. Correlation is significant at the 0.01 level (2-tailed).

Table S5 *Correlations Between Variables at the Participant Level in Study 2*

		SDO_scale_mean	warmth_mean	competence_mean	morality_mean	ind_id_mean	identity indispensability	economic indispensability	adoption_mean
SDO_scale_mean	Pearson Correlation	1	-,046	,028	-,035	-,401**	-,371**	,419**	-,278**
	Sig. (2-tailed)		,589	,737	,682	<,001	<,001	<,001	<,001
	N	143	143	143	143	143	143	143	143
warmth_mean	Pearson Correlation	-,046	1	,045	-,037	,095	-,019	-,053	,063
	Sig. (2-tailed)	,589		,595	,657	,261	,826	,532	,451
	N	143	143	143	143	143	143	143	143
competence_mean	Pearson Correlation	,028	,045	1	,065	,061	,136	-,127	-,013
	Sig. (2-tailed)	,737	,595		,441	,468	,105	,130	,874
	N	143	143	143	143	143	143	143	143
morality_mean	Pearson Correlation	-,035	-,037	,065	1	,015	-,012	,054	,152
	Sig. (2-tailed)	,682	,657	,441		,863	,883	,523	,070
	N	143	143	143	143	143	143	143	143
identity indispensability	Pearson Correlation	-,401**	,095	,061	,015	1	,885**	-,109	,680**
	Sig. (2-tailed)	<,001	,261	,468	,863		<,001	,195	<,001
	N	143	143	143	143	143	143	143	143
economic indispensability	Pearson Correlation	-,371**	-,019	,136	-,012	,885**	1	-,136	,706**
	Sig. (2-tailed)	<,001	,826	,105	,883	<,001		,105	<,001
	N	143	143	143	143	143	143	143	143
own_maintenance_mean	Pearson Correlation	,419**	-,053	-,127	,054	-,109	-,136	1	-,002
	Sig. (2-tailed)	<,001	,532	,130	,523	,195	,105		,980
	N	143	143	143	143	143	143	143	143
adoption_mean	Pearson Correlation	-,278**	,063	-,013	,152	,680**	,706**	-,002	1
	Sig. (2-tailed)	<,001	,451	,874	,070	<,001	<,001	,980	
	N	143	143	143	143	143	143	143	143

**. Correlation is significant at the 0.01 level (2-tailed).

Figure S1. *The Interaction between Morality and Warmth in Study 2*

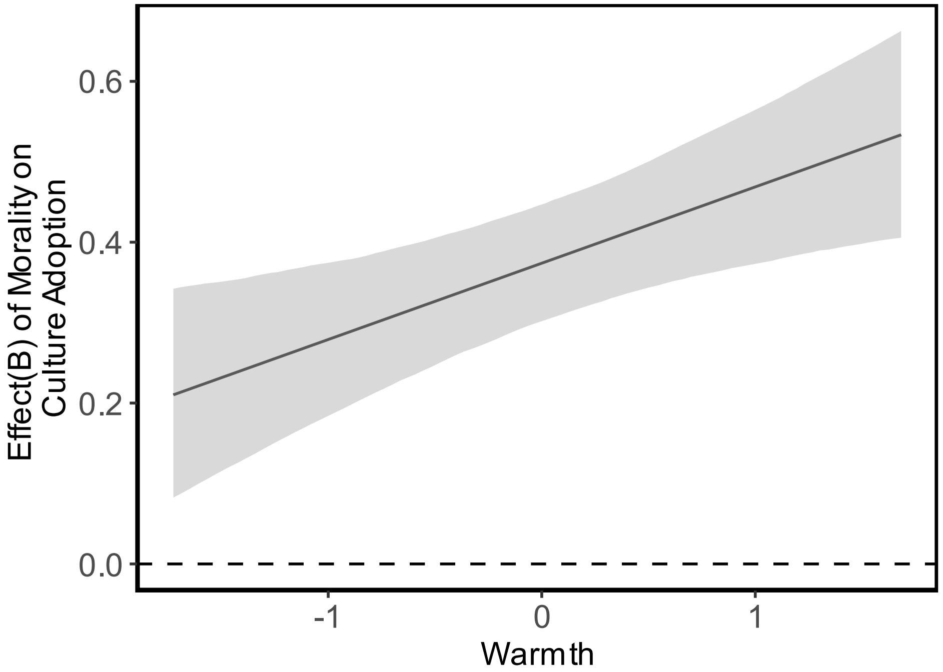

Table S6. *Correlations Between Variables at the Trial Level in Study 3*

		identity indispensability	economic indispensability	culture adoption
identity indispensability	Pearson Correlation	1	-,023	,201[**]
	Sig. (2-tailed)		,538	<,001
	N	730	730	730
economic indispensability	Pearson Correlation	-,023	1	,175[**]
	Sig. (2-tailed)	,538		<,001
	N	730	730	730
adoption	Pearson Correlation	,201[**]	,175[**]	1
	Sig. (2-tailed)	<,001	<,001	
	N	730	730	730

**. Correlation is significant at the 0.01 level (2-tailed).

Study 1: SDO Analysis

As pre-registered, we explored whether SDO moderated the main effects of the three dimensions of intergroup perception. High SDO individuals may selectively adopt high-status immigrant culture to maintain dominance, rejecting perceived low-status cultures (i.e., those scoring low on the dimensions), or conversely, could resist adopting both low and high-status cultures altogether to uphold the social hierarchy (Sidanius & Pratto, 2012).

Methods

Participants completed the 8-item (e.g., "An ideal society requires some groups to be on top and others to be on the bottom.") short form of the SDO-7 scale (α = .87; Ho et al., 2015). Responses were recorded on a 7-point Likert scale ranging from 1 (*strongly oppose*) to 7 (*strongly favor*).

Results

Finally, as pre-registered, we explored whether the effects of the three intergroup perception dimensions on culture adoption would be moderated by SDO. Indeed, SDO moderated the quadratic effect of competence (see Table S7). As displayed in Figure S2, for participants with medium SDO, the association of competence on culture adoption was positive and linear. However, the association was curvilinear for participants low and high in SDO, yet in divergent ways. For those high in SDO, cultural adoption was especially low for groups rated as low in competence, and the positive association of competence flattened out and even decreased somewhat after the midpoint of the scale. For those low in SDO, the shape of the slope was the opposite, with cultural adoption being highest for groups rated low and high in competence.

Table S7. *Linear Mixed Model Results Testing Whether the Association Between the Intergroup Perception Dimensions and Other Culture Adoption was Moderated by SDO in Study 1*

	B	*SE*	*df*	*t*	*p*
(Intercept)	2.66	0.20	204.04	13.27	< .001
SDO	-0.16	0.07	199.51	-2.38	.018
Warmth	15.14	3.66	102.57	4.14	.000
Warmth^2	-0.33	2.76	2367.10	-0.12	.905
Competence	-0.53	3.37	1487.71	-0.16	.876
Competence^2	6.70	2.75	2565.95	2.43	.015
Morality	5.58	4.36	624.42	1.28	.201
Morality^2	3.89	3.02	2411.98	1.29	.198
SDO x Warmth	-1.95	1.04	2591.44	-1.87	.062
SDO x Warmth^2	1.39	0.90	2597.45	1.54	.123
SDO x Competence	1.19	1.10	2584.85	1.08	.282
SDO x Competence^2	-2.37	0.90	2567.49	-2.63	.009
SDO x Morality	1.12	1.42	2585.15	0.79	.432
SDO x Morality^2	-0.88	1.01	2574.34	-0.88	.379

Figure S2. *Curvilinear Effect of Competence on Culture Adoption at Low, Medium, and High Levels of SDO in Study 1*

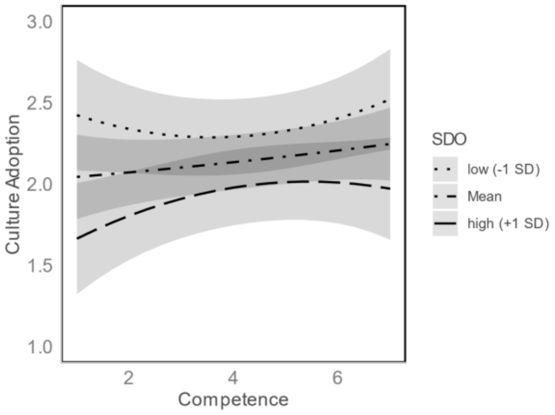

Note. Ribbons represent 95% confidence intervals.

Study 2: SDO Analysis

As in Study 1, we also explored the role of intergroup perceptions in moderating the relationship between own culture maintenance and other culture adoption. Moreover, we examined whether SDO would moderate the influence of the intergroup perceptions on other culture adoption.

Methods

As in Study 1, participants first completed the short-form SDO scale (α = .88).

Results

As in Study 1 and as pre-registered, we explored whether SDO would moderate the effects of the intergroup perception factors on other culture adoption. However, none of the interactions reached significance, *ps* > .328. Also, the relationship between own culture maintenance and other culture adoption was not significantly moderated by intergroup perceptions, *ps* > .358.

Tests of Curvilinearity

Whereas our primary interest lay in the linear main effects of these three intergroup perceptions, we also explored potential curvilinearity and interactions. One potential non-linear trend is that culture adoption rises steadily up to the midpoint of scales assessing warmth, competence, and morality, and then the growth rate plateaus. This pattern would mean that the evaluation of immigrant groups only needs to reach a level of warmth, competence, and morality at which evaluations become generally positive, after which further increase on these dimensions stops making a meaningful difference. Alternatively, culture adoption may be low both when immigrants are perceived to be very low or very high in warmth, competence, and morality, since both low and too high values may be perceived as undesirable. Low levels may indicate a contamination of the majority-group members' culture, whereas high levels may elicit negative perceptions of immigrants as superior to the majority group.

Study 1: Test of Quadratic Effects

We tested whether these effects might be better represented as quadratic. When we added quadratic terms to the regression model, R^2 (fixed effects) = 0.08, R^2 (total) = 0.72 (see Table S8), only the quadratic effect of warmth reached significance. As visualized in Figure S3, the slope of warmth was relatively flat until the scale's midpoint and then accelerated.

Table S8 *Linear Mixed Model Results Testing Main (Step 1) and Quadratic (Step 2) Associations with Cultural Adoption in Study 1*

	B	95% *CI*		*t*	*df*	*p*
(Intercept)	2.23	2.06	2.39	26.21	165.19	<.001
Warmth	9.91	5.43	14.39	4.34	15.46	.001
Competence	3.17	0.18	6.15	2.08	170.478	.039
Morality	8.70	4.91	12.50	4.50	29.97	<.001
Warmth^2	3.38	0.82	5.95	2.58	875.37	.010
Competence^2	0.00	-2.40	2.39	0.00	1753.21	.998
Morality^2	1.64	-0.86	4.14	1.28	1339.09	.200

Figure S3

Quadratic Relationship Between Warmth and Culture Adoption in Study 1

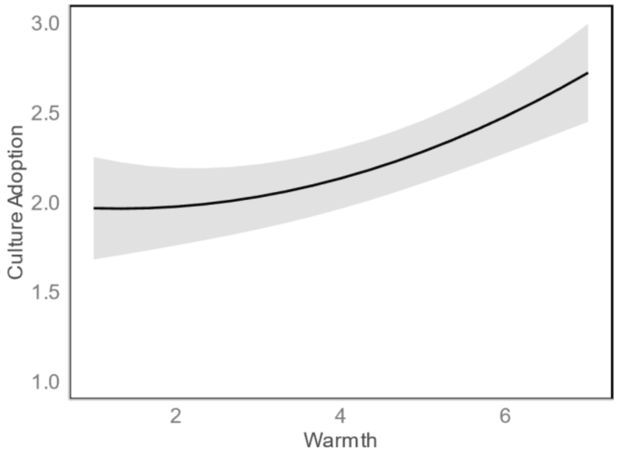

Note. Ribbons represent 95% confidence intervals.

Whereas the effects of competence and morality were best represented as linear, the effect of warmth was curvilinear. Specifically, majority-group members' receptiveness toward the culture of immigrants increased when immigrants were rated above the midpoint of the warmth scale, arguably signaling low threat potential (Constantin & Cuadrado, 2021).

Study 2: Test of Quadratic Effects

Competence had a curvilinear impact in the second model, see Table S9, R^2 (fixed effects) = 0.11, R^2 (total) = 0.77. Note, however, that this effect fell just above the significance threshold. As presented in Figure S4, other culture adoption increased until about the mean of the competence scale, after which the curve flattened out.

Table S9

Linear Mixed Model Results Testing Main (Step 2) and Quadratic (Step 2) Effects on Other Cultural Adoption in Study 2

	B	95% *CI*		*t*	*df*	*p*
(Intercept)	3.23	3.01	3.45	28.42	104.336	<.001
Warmth	8.81	7.16	10.46	10.45	33.114	<.001
Competence	3.79	0.68	6.90	2.39	4.252	.071
Morality	9.98	8.01	11.95	9.94	5.905	<.001
Warmth^2	-0.67	-2.31	0.96	-0.81	576.865	.419
Competence^2	-1.63	-3.24	-0.01	-1.97	578.480	.049
Morality^2	-1.10	-2.76	0.55	-1.31	578.495	.191

Figure S4. *Quadratic Relationship Between Competence and Other Culture Adoption in Study 2*

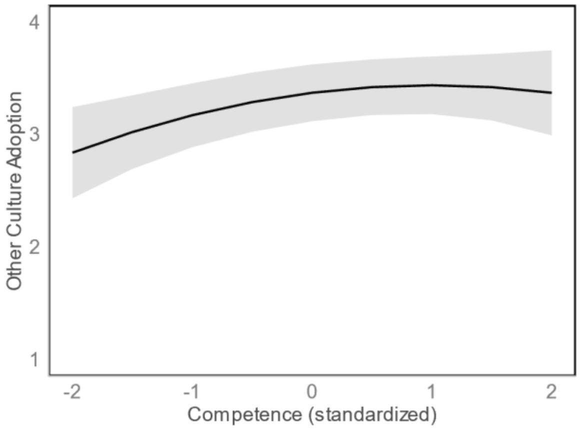

Note. Ribbons represent 95% confidence intervals.

Whereas the effect of warmth was curvilinear in Study 1, this time, the effect of competence was curvilinear. Specifically, competence led to more culture adoption, especially in the lower percentiles of the competence distribution, but then flattened out. Focusing on existing immigrant groups in Study 1 versus unnamed groups in Study 2 may have created distinct responses. Differences in the first study may therefore be partly attributable to participants’ existing biases or preconceptions about real-life immigrant groups

that may have intersected with and influenced the impact of perceived warmth and competence. Focusing on unnamed groups in this second study may have mitigated these inherent biases, allowing for a clearer understanding of the relationship between competence and other culture adoption. One potential interpretation of the finding may be that majority-group members are more open to adopting cultures that they perceive as neither incompetent nor too competent, possibly because this makes them feel less threatened by them as competitors (Fiske et al., 2002). However, as competence becomes relatively high, the motivation to adopt the culture plateaus, possibly because a perceived threat or emotions such as envy begin to overshadow the attractiveness of the culture's high competence (Cuddy et al., 2008; Komisarof et al., 2020)
